# PROM2 overexpression induces metastatic potential through epithelial‐to‐mesenchymal transition and ferroptosis resistance in human cancers

**DOI:** 10.1002/ctm2.1632

**Published:** 2024-03-21

**Authors:** Justine Paris, Claire Wilhelm, Celeste Lebbé, Mohammed Elmallah, Frédéric Pamoukdjian, Audrey Héraud, Guillaume Gapihan, Aurore Van De Walle, Van Nhan Tran, Diaddin Hamdan, Clara Allayous, Maxime Battistella, Emmanuel Van Glabeke, Kah Wai Lim, Christophe Leboeuf, Sébastien Roger, Géraldine Falgarone, Anh Tuan Phan, Guilhem Bousquet

**Affiliations:** ^1^ Université Paris Cité, INSERM, UMR_S942 MASCOT Paris France; ^2^ Laboratoire Physico Chimie Curie, Institut Curie, CNRS PSL Research University Paris France; ^3^ Université Paris Cité, INSERM Paris France; ^4^ APHP, Dermatolo‐Oncology Hôpital Saint Louis Paris France; ^5^ Inserm U1327 ISCHEMIA Université de Tours, Faculté de Médecine Tours France; ^6^ APHP, Hôpital Avicenne, Médecine Gériatrique Bobigny France; ^7^ Université Sorbonne Paris Nord Villetaneuse France; ^8^ School of Physical and Mathematical Sciences Nanyang Technological University Singapore Singapore; ^9^ Hôpital La Porte Verte, Cancérologie Versailles France; ^10^ Pathology Department APHP, Hôpital Saint Louis Paris France; ^11^ Fédération d'Urologie de Seine‐Saint‐Denis, CHI Robert Ballangé Aulnay‐sous‐Bois France; ^12^ APHP Hôpital Avicenne, Unité de Médecine Ambulatoire (UMA) Bobigny France; ^13^ NTU Institute of Structural Biology Nanyang Technological University Singapore Singapore; ^14^ APHP Hôpital Avicenne, Oncologie médical Bobigny France

**Keywords:** breast cancer, epithelial‐to‐mesenchymal transition, ferroptosis resistance, melanoma, metastases, prominin‐2, renal cancer

## Abstract

**Introduction:**

Despite considerable therapeutic advances in the last 20 years, metastatic cancers remain a major cause of death. We previously identified prominin‐2 (PROM2) as a biomarker predictive of distant metastases and decreased survival, thus providing a promising bio‐target. In this translational study, we set out to decipher the biological roles of PROM2 during the metastatic process and resistance to cell death, in particular for metastatic melanoma.

**Methods and results:**

Methods and results: We demonstrated that PROM2 overexpression was closely linked to an increased metastatic potential through the increase of epithelial‐to‐mesenchymal transition (EMT) marker expression and ferroptosis resistance. This was also found in renal cell carcinoma and triple negative breast cancer patient‐derived xenograft models. Using an oligonucleotide anti‐sense anti‐PROM2, we efficaciously decreased PROM2 expression and prevented metastases in melanoma xenografts. We also demonstrated that PROM2 was implicated in an aggravation loop, contributing to increase the metastatic burden both in murine metastatic models and in patients with metastatic melanoma. The metastatic burden is closely linked to PROM2 expression through the expression of EMT markers and ferroptosis cell death resistance in a deterioration loop.

**Conclusion:**

Our results open the way for further studies using PROM2 as a bio‐target in resort situations in human metastatic melanoma and also in other cancer types.

## Graphical abstract

1. In human metastatic melanoma, PROM2 is the cornerstone of two cancer hallmarks: the metastatic process and resistance to ferroptotic cell death.
2. The invasive and migration phenotypes linked to PROM2 overexpression are associated with an EMT phenotype.
3. The combination of these two cancer hallmarks, by way on an aggravation loop, contributes to increasing the metastatic burden, leading to death.

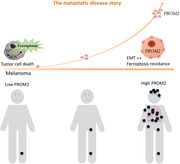

## 1 INTRODUCTION

Despite considerable therapeutic advances in the last 20 years, metastatic cancers remain the major cause of death, because patients almost always develop resistances to initially efficacious treatments. Metastatic melanoma is no exception to this rule. Immunotherapies and targeted therapies have transformed patient prognosis, with very long survivors.^1^, ^2^ However, approximately 50% of patients remain non‐responsive to these treatments or develop secondary resistance.^3^ Therefore, it appears necessary to identify new biotargets for treatment. In a series of 101 melanoma lymph node metastases, using transcriptomic analyses on laser‐microdissected melanoma cells, we identified prominin‐2 (PROM2) as a biomarker predictive of distant metastases and decreased survival, thus providing a promising new bio‐target.^4^

PROM2 is a 112 kDa glycoprotein composed of five transmembrane segments and two large glycosylated extracellular loops.^5^ It is a paralogue of PROM1 (CD133), with only 32% of the amino acid identity. Both are cholesterol‐binding proteins and they are physiologically associated with plasma membrane protrusions. In humans, *PROM2* mRNA is physiologically expressed in various normal tissues, but its expression seems restricted to epithelial cells.^6^ PROM2 is distributed in non‐polarised manner between the apical and basolateral plasma membranes. However, its expression is higher in cilia, microvilli and other acetylated tubulin‐positive protruding structures.^6^ In addition, PROM2 is involved in the regulation of caveolar endocytosis, and its overexpression leads to significant changes in plasma membrane organisation and function, including an increase of protrusions, and thus of invasive potential.^7^, ^8^

Physiologically, PROM2 has recently been described as a ferroptosis inhibitor.^9^ Ferroptosis is a form of regulated cell death characterised by iron‐dependent lipid peroxidation.^10^ Typically, cell detachment from the extracellular matrix is a ferroptotic stimulus.^11^, ^12^ However, under physiological or pathological conditions, detached cells can express pro‐survival factors, including PROM2 which enables the formation of ferritin‐containing multivesicular bodies, in turn responsible for iron transport out of the cell, thus inhibiting ferroptosis.^9^

Overall, considering previously published data including ours, PROM2 could be the cornerstone of two cancer hallmarks in human metastatic melanoma^13^, ^14^, ^15^: the metastatic process and resistance to cell death.

In this translational study, we set out to decipher the biological roles of PROM2 during the metastatic process, in particular for metastatic melanoma. Both in vitro and in vivo, we successively demonstrated that PROM2 overexpression increased invasion and migration, and thus the metastatic process via epithelial‐to‐mesenchymal transition (EMT) activation, and that PROM2 overexpression was associated with ferroptosis resistance in melanoma. Using a new murine model, we showed that the combination of these two hallmarks, by way on an aggravation loop, contributed to increasing the metastatic burden.

## 2 METHODS

### 2.1 Human samples

Formalin‐fixed tissue samples from patients with metastatic melanoma or metastatic clear‐cell renal carcinoma were used in this study (see Figure S1). In compliance with French bioethics law (2004‐800; 8 June 2004), all patients had been informed of the research use of the part of their samples remaining after diagnosis had been established, and none opposed it. Informed consent was obtained from each patient.

### 2.2 Melanoma cell lines

The human melanoma A375 cell line was purchased from Sigma–Aldrich (#88113005; Merck, Germany) and the Sk‐Mel‐28 cell line was purchased from American Type Culture Collection (#HTB72; ATCC, USA). The cell lines were cultured in a humidified atmosphere containing 5% CO_2_ at 37°C. The cells were grown in Dulbecco's modified Eagle medium (DMEM) with high glucose content (#11965084; Gibco, France) supplemented with 1% of penicillin/streptomycin (#P4458; Sigma–Aldrich, France) and 10% horse serum (#16050122; Gibco).

### 2.3 Modulation of *PROM2* in melanoma cell lines

For *PROM2* overexpression, 4 × 10^5^ A375 or Sk‐Mel‐28 cells were seeded into six‐well plates to reach 70% confluence. The following day, cells were transfected with the human PROM2 activation kit by crispra (#GA115763; Origene, Germany) using Lipofectamine 3000 (#L3000001; Invitrogen, France), according to the manufacturer's instructions. After 1 week, a limit dilution was performed to obtain several clones. *PROM2* over‐expression was then confirmed using RT‐qPCR. A375 and Sk‐Mel‐28 cells overexpressing *PROM2* were named A375 PROM2 and Sk‐Mel‐28 PROM2, respectively.

For *PROM2* knock‐out, 4 × 10^5^ A375 or Sk‐Mel‐28 cells were seeded into six‐well plates to reach 70% confluence. The following day, cells were transfected with PROM2 human gene knock‐out kit by crispr (#KN411605; Origene) using Lipofectamine 3000. After 48 h, knock‐out cells were selected using an increasing dose of puromycin to kill all non‐transfected cells. A limit dilution was then performed to obtain several KO clones. Decreased expression of PROM2 was confirmed using RT‐qPCR. The *PROM2* knock‐out cells were named A375 KO and Sk‐Mel‐28 KO.

### 2.4 In vitro cell proliferation assay

For each cell line, transfected or not, 6000 cells were seeded in 96‐well plates, and then counted every day for 5 consecutive days using KOVA slides. Each experiment was performed in triplicate.

### 2.5 In vitro cell migration and invasion assays

Cell migration was assessed using Boyden chambers on 12‐well plates, with a cell culture insert of 10.3 mm diameter and membrane pores of 8 µm (#PCEP12H48; Becton Dickinson, France). For the invasion assays, we used Boyden chambers coated with Matrigel (#356237; Falcon, MA, USA). Migration and invasion were assessed for each cell line, transfected or not. For each experiment, 3 × 10^4^ cells were seeded in the upper chamber in a medium without horse serum, and the lower chambers were filled with high glucose DMEM medium supplemented with 10% horse serum at 37°C in a 5% CO_2_ atmosphere.

After 24, 48 and 72 h of incubation, cells on the upper face were removed and cells on the lower face of the filter were fixed, stained with crystal violet (#HT90132; Sigma–Aldrich, USA) and counted. Ten fields were analysed on each filter, and results were expressed as means ± standard error of the mean (SEM). Each experiment was performed in triplicate.

### 2.6 In vitro spheroid invasion assay

Spheroids from the different cell lines were generated in agarose microwells with a diameter and depth of 200 µm. The microwells were fabricated directly within each well on a 96‐well multiplate by pouring in 50 µL of hot 2% agarose (#A6877; Sigma–Aldrich) and then placing a 3D printed stamp that has 50 pillars of 200 µm diameter and height. Agarose solidifies in less than 5 min at room temperature, the stamp is then pulled out of the well, with the fabricated agarose microwells. The cells were centrifuged in the 200 µm agarose microwells at a density of 200 cells per well. Each cell line resulted in the formation of spheroids after 1 day, which grew over the following days.

For the invasion assay, 50 µL per well of 2 mg/mL cold collagen (#354236; Corning, France) was poured on the spheroid pattern formed, then placed at 37°C for gelation. 100 µL of complete medium was then added to the wells. Images of spheroid invasion were captured immediately after collagen addition, as well as at 24 and 48 h later. The invasion area was quantified at the 24‐h time point through image analysis, expressed in µm^2^. Image acquisition was performed using an EnSight® Multimode Microplate Reader (PerkinElmer) coupled with the Kaleido 3.0 software. Subsequently, ImageJ software was employed for image processing. In summary, the image taken 24 h after collagen addition underwent thresholding, binarisation and subsequent measurement of the area.

### 2.7 Cell viability assay

For cell viability assessment, A375 or Sk‐Mel‐28 cell lines were seeded separately in 96‐well tissue culture plates at a density of 5000 cells per well. After 24 h of incubation, the cells were exposed to increasing concentrations of RSL3 or erastin for 24 additional hours. Cell viability was determined by the colorimetric conversion of yellow, water‐soluble tetrazolium MTT (3‐[4,5‐dimethylthiazol‐2‐yl]−2,5‐diphenyl‐tetrazolium‐bromide; #475989; Sigma), to purple, water‐insoluble formazan. After incubation for 2 h at 37°C with 0.4 mg/mL of MTT, the absorbance was measured at 560 nm using a microplate reader (ThermoFisher, France). Experiments were performed in triplicate, and untreated cells were used as positive controls and medium without cells as a negative control.

### 2.8 Cell detachment assay

For each cell line, 4 × 10^6^ cells were seeded into cell‐repellent surface six‐well plates (#657970; Greiner Bio‐One, Germany) in serum‐free medium. After 30, 60 or 120 min, cells were retrieved and *PROM2* mRNA expression was assessed using RT‐qPCR.

### 2.9 Patient‐derived xenografts

These have been previously described in several articles.^16^, ^17^, ^18^, ^19^ Female nu/nu athymic mice of NMRI (R. Janvier, France) background, aged 5–8 weeks, were used as xenograft recipients for human melanoma samples. The National Ethics Committee for experimental animal studies approved this study (APAFIS#17190‐2018101814245111). The ethics guidelines that were followed met the standards required by the US guidelines.^20^ Animals were maintained in the pathogen‐free housing at Sorbonne Paris Nord University (agreement number: C9300801). For the initial xenograft, 5 mm^3^ of human tumour fragments were grafted subcutaneously in 5–10 mice under xylasin (10 mg/kg body weight) and ketamin anaesthesia (100 mg/kg body weight). For each further round, 10 mm^3^ fragments were xenografted into five mice.

A clinical score was assessed daily and tumour growth measured in two perpendicular diameters with a calliper. Tumour volume was calculated as *V* = *L* × *l* ^2^/2, *L* being the larger diameter (length) and *l* the smaller (width).^21^, ^22^ The mice were euthanised when the tumour weight reached the ethically recommended limit of less than 10 % of mouse weight (Directive 2010/63/EU of European Parliament and Council of 22 September 2010 on the protection of animals used for scientific purposes; Official Journal of European Union L 276/33).

For each mouse, the tumour as well as different organs were systematically analysed. After dissection, each tumour was cut into three parts: one part was immediately snap‐frozen in liquid nitrogen, one part was formalin‐fixed and paraffin‐embedded, and a third part was used for the new passage. Each organ was analysed using haematoxylin–eosin stained paraffin‐embedded tissue sections.

### 2.10 Metastatic models of melanoma cell lines

Female nu/nu athymic mice of NMRI background were intravenously injected with either A375, A375 PROM2, Sk‐Mel‐28 or Sk‐Mel‐28 PROM2 5 × 10^6^ cells in 100 µL of NaCl. A clinical score was allocated daily and the mice were euthanised at 6 weeks, or earlier if ethically required (clinical signs of suffering, weight loss). Particularly, the weight was checked twice a week and clinical signs suggestive of complications related to metastases were monitored daily (abnormal breathing/rapid respiratory movements, abnormal position of the back, locomotion disorder, increased abdomen volume linked to ascites).

Each mouse was monitored weekly using ultrasound sonography (Aplio XG SSA‐790A; Toshiba, France). We used two different ultrasound probes, one at 9–12 MHz for deep structure evaluation (liver, abdomen, lung), and one at 18 MHz for superficial structure evaluation (muscles). For each mouse, under superficial anaesthesia using isoflurane, a systematic evaluation was done by sector, except brain which exploration is limited by the presence of the skull that prevent from ultrasound diffusion. This evaluation was performed by a medical practitioner with expertise in ultrasound imaging (GF), in order to monitor metastases in mice, mainly lung, muscle or abdominal localisations. Ultrasound imaging enables to detect early metastases, mainly unexpected localisations to avoid brutal death. For lung localisations, the method was previously reported in patients by our research team.^23^

After euthanasia, an assessment of metastatic extension in the lung was performed on haematoxylin–eosin stained tissue sections. Areas of lung metastases were delineated and quantified using DotSlide software (Olympus, Tokyo). Then the mean surface area of metastatic extension was calculated for all animals in each group.

### 2.11 mRNA gene expression quantification

For all cell lines, total RNA was extracted from 10^6^ viable cells. For tumour xenografts, it was extracted from 20 frozen sections of 5 µm. Total RNA was extracted using RNeasy‐Mini‐Kit (#74104; Qiagen, France) and quantified on NanoDrop (LabTech, France). Human *PROM2* gene expression was assessed using the human *PROM2* primer Hs00376331_m1 (ThermoFisher). EMT gene expression was assessed using human *ZEB1* primers (Hs00232783_m1), *ZEB2* (Hs00207691_m1), *SNAI1* (Hs00195591_m1), *SNAI2* (Hs00950344_m1), *TWIST1* (Hs01675818_s1), *TWIST2* (Hs02379973_s1), *CDH1* (Hs01023894_m1) and *VIM* (Hs00185584_m1) (ThermoFisher). Human *CAV1* gene expression was assessed using the human *CAV1* primer Hs00971716_m1 (ThermoFisher). Total RNA was reverse‐transcribed before qPCR amplification using random primers with GoScript™ Reverse Transcriptase (#A5001; Promega, France). The qPCR reactions were performed using fluorescent probes on a CFX96 Real Time System (Bio‐Rad, France). A blank sample (no cDNA) was included and the experiments were performed in triplicate for each gene. The housekeeping genes *TBP* (Hs00427620_m1) and *GAPDH* (Hs02758991_g1) were used to normalise gene expression results. Each RT‐qPCR assay was performed according to the MIQE guidelines^24^ and conducted in triplicate.

### 2.12 Droplet digital PCR assay

ddPCR assay was used to quantify PROM2 and EMT marker mRNA expression in laser‐microdissected cancer cells on tissues (primary tumour xenografts, metastatic localisations of murine models and patients).

For each tumour sample, 7 µm‐thick tissue sections were laser‐microdissected to select a minimum of 300 tumour cells for a minimum surface area of 0.043 mm^2^, using a PALM‐Microbeam/Zeiss‐system (Carl Zeiss, Germany).

Droplet digital polymerase chain reaction (ddPCR) was performed using the QX100 ddPCR workflow system (Bio‐Rad, Hercules, USA). The mix contained 20 ng of genomic RNA, 10 µL of One‐Step RT‐ddPCR kit for Probes (#1864021; Bio‐Rad), 1 µL of probes (either *PROM2*, *ZEB1*, *SNAI1* or *TWIST1*) and 1 µL of *GAPDH* probes per well, and the final volume for the reaction was 20 µL. Droplets were generated by a QX200 Droplet Generator (Bio‐Rad). PCR was carried out on the CFX96 Real Time System (Bio‐Rad). PCR was performed with an initial denaturing step at 95°C for 10 min, followed by 40 cycles of denaturing (94°C for 30 s), and annealing (60°C for 1 min). A post‐amplification melting curve program was initiated by heating to 98°C for 10 min and then cooling down to 12°C. Each PCR run included a no‐template control. The results of ddPCR were generated using QX100 Droplet Reader (Bio‐Rad) and analysed using QuantaSoft software (Bio‐Rad). The ratio of positive droplets to GAPDH‐positive droplets was calculated.

### 2.13 Cell and tissue immuno‐staining

For immunofluorescence staining, cell lines were grown separately on culture slides (#354104; BD Falcon™, BD Biosciences). Then, an indirect immunofluorescence method was run using anti‐human PROM2 monoclonal antibody (#TA500350, Clone 13A9, 1/50; Origene, USA) as the primary antibody. Staining was performed with Tyramide detection kit 488CF (#33001; Biotium, USA), and fluorescent mounting medium with DAPI was used for nucleus detection (E19‐18; GBI labs, USA). The fluorescence staining was observed at 400× magnification on a BX63 microscope (Olympus).

For immunohistochemistry on tissues sections, an indirect immunoperoxidase method was applied on 5 µm‐thick tissue sections using anti‐human PROM2 monoclonal antibody (#TA500350, Clone 13A9, 1/50; Origene), anti‐ZEB1 antibody (ab203829, 1/100; Abcam, UK) and anti‐SNAIL+SLUG antibody (ab180714, 1/100; Abcam) as primary antibodies. For PROM2 staining, the antibody was directly biotinylated using Mix‐n‐stain Biotin Antibody Labelling Kit (MXBIOS20; Merck, Germany). For ZEB1 and SNAIL+SLUG staining, the primary antibodies were coupled with anti‐rabbit OmniMap detection kit (#05269679001; Roche Diagnostic, Meylan, France). The systematic controls used were absence of primary antibody and use of an irrelevant primary antibody of the same isotype. For PROM2 staining, each sample was given a score as previously described^4^ by multiplying the staining intensity grade (0 = no staining, 1 = low intensity, 2 = medium intensity, 3 = strong intensity) by the numerical code for the percentage of positive cells (0 = 0%, 1 = under 10%, 2 = 10−50%, 3 = 51−80%, 4 = over 81%). The results were expressed as means ± SEM. For ZEB1 and SNAIL+SLUG staining, antibodies expressing cells were counted on five different fields at ×200 magnification. A BX63 microscope (Olympus) with wide‐field eyepiece number 26.5 was used, providing a field size of 0.344 mm^2^ of tumour cells. Results were expressed as means ± SEM.

### 2.14 Ferrous iron quantification

Ferrous iron quantification was used to assess ferroptotic pathway.

For each cell line, 4 × 10^6^ A375 or Sk‐Mel‐28 cells were equally seeded into six‐well plates. The cells were treated with RSL3 (0.5 µM, #SML2234; Sigma), erastin (5 µM, # E7781; Sigma) and deferoxamine (5 µM, DFO, #D9533; Sigma) for 24 h. RSL3 and erastin concentrations were chosen according to previously published data on A375 cell lines.^25^ To confirm their biological relevance, we have done a MTT assay for the three cell lines (A375 KO, A375 and A375 PROM2). In particular, for A375 KO, IC_50_ was 5 µM for erastin and 0.5 for RSL3 (Figure S2). For DFO, we purposely chose a concentration lower than 10 µM, not to induce cell death while inhibiting ferroptosis.^26^

For tumour xenografts, total proteins were extracted and concentrations were measured by spectrophotometry using Pierce BCA Protein Assay Kit (#5000001; Bio‐Rad). For each sample, ferrous iron was quantified in 150 µg of total proteins using an Iron Assay Kit (#MAK025; Sigma–Aldrich) according to the manufacturer's instructions. In this assay, iron is released by the addition of an acidic buffer. Released iron is reacted with a chromagen resulting in a colorimetric (593 nm) product, proportional to the iron present. The values obtained from the appropriate iron standards were used to plot a standard curve. Concentration of Iron Sa/Sv = C, Sa = amount of iron in unknown sample (nmole) from standard curve and Sv = sample volume (µL) added into the wells. Iron atomic mass being 55.85 g/mole, concentration of ferrous iron in sample is calculated as *C* × 55.85 and is expressed in ng/mL.

For spheroids, ferrous iron was detected by fluorescence staining. The spheroids, grown in microwells as reported above were incubated at 37°C for 1 h with FerroOrange solution (#SCT210; BioTracker™ FerroOrange, Millipore) at a concentration of 2 µM, then washed twice with HBSS buffer to remove any extracellular Fe^2+^. BioTracker FerroOrange Live Cell Dye is an orange fluorescent probe that specifically detects labile Fe^2+^ only. The intensity of fluorescence does not increase in the presence of Fe^3+^ or bivalent metal ions other than iron. The fluorescence was finally observed fluorescence (542 nm/572 nm) and expressed as a relative fluorescence intensity.

### 2.15 Lipid peroxidation quantification

Lipid peroxidation (4‐HNE) was quantified to assess a final step of ferroptotic pathway.

For each cell line and for the xenografts, cells were lysed using RIPA lysis buffer (#89900; Thermo Scientist, USA) and total protein concentration was measured by spectrophotometry using Pierce BCA Protein Assay Kit (#5000001; Bio‐Rad). Lipid peroxidation was quantified in 100 µg of protein using Lipid Peroxidation (4‐HNE) Assay Kit (ab238538; Abcam) according to manufacturer's instructions: a plate was coated overnight with 4‐HNE conjugate, samples and anti‐4HNE antibody were added to the wells, then a secondary antibody horseradish peroxidase (HRP) conjugate was added and finally the substrate solution. Lipid peroxidation was measured at 450 nm.

### 2.16 Western blot for protein quantification

Western blot analyses were performed to quantify protein expression of various markers, including PROM2, EMT markers, CAV1, 4‐HNE and TGS101.

Analyses were performed on each cell line, and on tumours from the five patient‐derived melanoma xenografts. Total proteins were extracted from 20 sections of 5 µm of frozen tumour samples using RIPA lysis buffer (#89900; Thermo Scientist) containing anti‐proteases and anti‐phosphatases (S8820‐20TAB and 4906845001; Sigma–Aldrich). Protein concentrations were measured by spectrophotometry using Pierce BCA Protein Assay Kit (#5000001; Bio‐Rad). Samples were diluted in distilled water and loading buffer to reach 30 µg, then heated at 95°C for 10 min for protein denaturation. Proteins were separated by electrophoresis on SDS‐page with 10% polyacrylamide gel (#4561033; Bio‐Rad), transferred to a nitrocellulose membrane (0.45 µm) and stained with Ponceau red. Non‐specific sites of the membranes were saturated with TBST containing 5% milk. The membranes were then incubated overnight at 4°C with one of the following primary antibodies: PROM2 (1/500, #NBP‐38032; Novus Bio, USA), ZEB1 (1/500, #ab203829; Abcam), SNAI/SLUG (1/1000, #ab85936; Abcam), VIM (1/1000, #PA527231; Thermo Scientist), TSG100 (1/1000, #SAB5700757; Sigma–Aldrich), CAV1 (1/1000, #PA517447; Thermo Scientist) or GAPDH (1/2500, #ab9485; Abcam) antibody. The membranes were then incubated for 1 h at room temperature with rabbit anti‐human IgG coupled with peroxidase (1/20000, #A16023; Invitrogen). Peroxidase activity was subsequently revealed with Clarity Western ECL (#5000116; Bio‐Rad). GAPDH was taken as a charge control. Chemiluminescence was observed using ChemiDoc (Bio‐Rad) and measured using Image Lab 6.1 version (Bio‐Rad). Briefly, draw regions of interest around each band are delineated, and the software measure the intensity. Then, the intensity of the protein bands of interest are normalised to an internal control or loading control (here GAPDH), to correct for variations in protein loading, transfer efficiency and other experimental variables. The intensity of each band is expressed as a ratio to the intensity of the loading control, which provides the normalised value.

### 2.17 Anti‐PROM2 antisense oligonucleotide synthesis

Several antisense oligonucleotides (ASOs) were designed to be complementary to *PROM2* mRNA (RefSeq ID NM_144707.4). All ASOs were synthesised in‐house with an ABI 394 DNA/RNA synthesiser using DNA and locked nucleic acid (LNA) phosphoramidites from Glen Research. The ASOs were fully phosphorothioate (PS)‐modified using phenylacetyl disulphide (#RN‐1496; ChemGenes Corporation, USA) as the sulphurising reagent. Sequences of the two selected PROM2 ASOs and control ASO are listed in Table 1. Cleavage from solid base and de‐protection of the ASOs were carried out using concentrated aqueous ammonia at 55°C for 16 h. The ASOs were subsequently purified using Poly‐Pak II cartridges (#60‐3100; Glen Research, USA) or HPLC, desalted using Glen Pak 2.5 desalting column (#61‐5025; Glen Research) and dried by lyophilisation. The ASOs were re‐suspended in phosphate‐buffered saline (PBS) before use. All ASOs were characterised by JEOL SpiralTOF matrix‐assisted laser desorption/ionisation time‐of‐flight mass spectrometer.

**TABLE 1:** ASO sequences used in this study.

Name	Sequence^a^	Target site^b^
*PROM2‐ASO1*	T * C * C *A*T*T*T*G*T*A*A*T*G* C * C * A	3806‐3821
*PROM2‐ASO2*	A * C * T *T*G*T*T*G*C*T*G*G*T* T * T * A	3610‐3625
*ASO‐control*	G * G * C *T*A*G*A*T*G*C*T*A*A* C * C * T	–

^a^ LNA nucleotides are underlined, and PS modifications are labelled with asterisks (*).

^b^ Position on PROM2 mRNA (RefSeq ID NM_144707.4).

For the in vitro experiments, cells were treated for 4 days with ASO concentrations ranging from 50 to 4000 nM.

For spheroid assays, ASO treatment was initiated in culture flasks 1 day before cell deposition into microwells. Two ASO concentrations, namely 50 and 500 nM, were chosen. On day 0, A375 PROM2 cells were suspended in complete medium supplemented with ASO at the specified concentrations, or without ASO, and subsequently centrifuged onto the microwells. By day 3, collagen was prepared with the corresponding ASO concentrations and deposited on top of the microwells. Additionally, 100 µL of complete medium, supplemented with ASO at concentrations of 50 and 500 nM, or without ASO, was added. Imaging was conducted immediately after collagen addition (time 0), as well as 24 and 48 h later. Image analysis was specifically performed at the 24‐h time point.

### 2.18 In vivo enrichment loop

First, five mice corresponding to round 0 were injected intravenously with 5 × 10^6^ A375 PROM2 cells diluted in 100 µL of NaCl. A clinical score was allocated daily and weekly ultrasound imagery was performed. The mice were euthanised at 6 weeks. At euthanasia, macroscopic metastases from different sites were taken up, filtered on a 70 µm filter and cultured in A375 cell line medium. *PROM2* mRNA expression was assessed using RT‐qPCR for each cultured cell. Those with the highest *PROM2* mRNA expression were reinjected intravenously into five new mice. These mice corresponded to round 1. This pattern was reproduced three times. Each mouse was monitored weekly using ultrasound imagery and euthanised at 6 weeks or earlier if ethically justified. At euthanasia, the metastatic extension was assessed in each organ on 2 µm slides on haematoxylin–eosin stained paraffin‐embedded tissue sections. The lung metastatic surface area was quantified for each mouse as described previously.

### 2.19 Exosome isolation and characterisation

#### 2.19.1 Materials

Antibodies for western blot, mouse anti‐Alix mAb (3A9), rabbit anti‐flotillin mAb (#3253) and rabbit anti‐Grp94 mAb (#2104) were purchased from Cell Signaling Technology (Massachusetts, USA). Mouse anti‐CD9 mAb (C‐4, sc‐13118), mouse anti‐CD81 (B‐11, sc‐166092), mouse anti‐CD63 mAb (MX‐49.129.5, sc‐5275), mouse anti‐TSG101 mAb (C‐2, sc‐7964) and mouse anti‐β‐actin mAb (C‐4, sc‐47778) were purchased from Santa Cruz Biotechnology (Heidelberg, Germany). Rabbit anti‐PROM2 mAb (NBP2‐38032) was obtained from Novus Biologicals (Abingdon, United Kingdom), anti‐human CD9 antibody‐PE (Clone REA1071), anti‐human CD81 antibody‐PE (Clone REA513) and anti‐human CD63 antibody‐PE (Clone REA1055) were purchased from Meltenyi Biotec SAS (Paris, France). Ultracentrifuge tubes were purchased from Beckman Coulter Life Science (Villepinte, France).

#### 2.19.2 Isolation of exosomes

Exosomes from A375 (WT‐EXO) and A375 PROM2 (Prom2‐EXO) melanoma cell lines were isolated by differential ultracentrifugation as previously described.^27^ Briefly, cells were cultured in DMEM supplemented with 10% exosome‐depleted FBS until reached 80% confluency at 37°C and 5% CO_2_. The conditioned media (CM) was collected and successively centrifuged at 300 and 2000×*g* (10 min each) at 4°C to remove both cell debris and dead cells. The supernatant was collected in new tubes following each centrifugation step. Afterwards, CM was centrifuged at 10 000×*g* for 40 min at 4°C and then the supernatant was recovered in new tubes and passed through 0.2 µm Whatman filter (Cytiva Life Science, USA) to remove macroparticles. To isolate exosomes, the supernatant was ultra‐centrifuged at 110 000×*g* for 90 min at 4°C. the supernatant was removed and the precipitated exosomes were washed by filtered PBS and ultra‐centrifuged for 70 min at 4°C. Finally, PBS was removed and exosomes were resuspended in fresh PBS for further analysis.

#### 2.19.3 Nanoparticle tracking analysis

Size distribution and concentration of exosomes were determined by nanoparticle tracking analyses (NTAs) on ZetaView (Particle Matrix, Germany) with 488 nm laser connected to a scattered filter.^28^ Briefly, exosome samples were diluted in PBS to a final volume of 1 ml (1:1000 dilutions). Following calibration with standard polystyrene nanoparticles, samples were injected in the instrument and the measurements were performed by scanning 11 cell position/cycle. To record videos, all parameters were adjusted under the following settings: focus: autofocus; camera sensitivity: 92.0; shutter: 100; scattering intensity: 4.0; cell temperature: 25°C. Analysis of the recorded videos were performed by ZetaView software (version 8.05.14 SP7) with specific analysis parameters: maximum particle size: 1000; minimum particle size: 10; minimum particle brightness: 30; camera: FpSec 15. To determine the relative percentage exosome subpopulations in terms of their CD9, CD81 and CD63 tetraspanins content by fluorescence‐based NTA (f‐NTA), 9 µL of exosomes was incubated with 1 µL of the tetraspanins antibodies (CD9, CD81 or CD63) conjugated to phycoerythrin (PE) at the recommended dilution from suppliers and then incubated at room temperature for 2 h in dark. The stained mixtures were diluted 1:8000 and measured in scattered mode using the aforementioned parameters. The measurements of CD9, CD81 and CD63 subpopulations in all samples were carried out under the fluorescence mode using lase wavelength 488 nm‐fluorescence 500 nm filter. The relative percentage of tetraspanins subpopulations was calculated as follow:

$$\begin{array}{ccc} & & \mathrm{Relative}\;\%\;\mathrm{of}\;\mathrm{tetraspsnins}\;\left(\mathrm{CD}9,\;\mathrm{CD}81\;\;\mathrm{or}\;\mathrm{CD}63\right)\\ & & \;=\frac{\mathrm{Conc}.\;\mathrm{Flourescence}\;\mathrm{mode}\;\left(500\;\mathrm{nm}\right)}{\mathrm{Conc}.\;\mathrm{scattered}\;\mathrm{mode}}\times 100 \end{array}$$

Data were analysed by GraphPad prism 9 software (Massachusetts, USA).

#### 2.19.4 Transmission electron microscopy

Cells were fixed in 4% paraformaldehyde, 1% glutaraldehyde (Sigma) in 0.1 M phosphate buffer (pH 7.2) for 24 h. Samples were then washed in PBS and post‐fixed by incubation with 2% osmium tetroxide (Agar Scientific, Stansted, UK) for 1 h. Afterwards, cells were fully dehydrated in a graded series of ethanol solutions and propylene oxide. Impregnation step was performed with a mixture of (1∶1) propylene oxide/Epon resin (Sigma) and then left overnight in pure resin. Samples were then embedded in Epon resin (Sigma) to polymerise for 48 h at 60°C. Ultra‐thin sections (90 nm) of these blocks were obtained with a Leica EM UC7 ultramicrotome (Wetzlar, Germany). Sections were stained with 2% uranyl acetate (Agar Scientific), 5% lead citrate (Sigma) and observations were made with a JEOL 1400 plus transmission electron microscope (JEOL, Tokyo, Japan).

#### 2.19.5 Western blotting

Cells and exosomes were lysed in radioimmunoprecipitation assay (RIPA) buffer (Pierce® 899000; Thermo Fisher Scientific) and protease cocktail inhibitor‐EDAT free (cOmplete Mini, #11836170001; Sigma–Aldrich). Protein concentration was determined by Pierc ™ BCA protein assay kit (#23225; Thermo Fisher Scientific). Approximately 20 µg protein was loaded on 10% SDS‐PAGE and then the separated proteins were blotted onto nitrocellulose membrane (Thermo Fisher Scientific). The membranes were washed three times with Tris buffered saline containing 0.1% of tween 20 (TBST) pH 6.5 for 10 min/washing step followed by masking with 5% BSA for 1 h. Membrane development was performed by incubating the primary antibodies (ALIX, CD9, CD81, CD63, flotillin‐1, TSG101, Grp94, promini‐2 and β‐actin) with the recommended dilution from suppliers overnight at 4°C. The membranes were washed three times (10 min each) with TBST and then the membranes were incubated with the corresponding secondary antibodies conjugated to HRP for 1 h at ambient temperature. Following washing steps with TBST, target proteins were detected using an enhanced chemiluminescence kit SuperSignal^Tm^ West Pico Plus (Thermoscientific, Rockford, USA).

### 2.20 Statistical analysis

Quantitative variables were expressed as means ± standard deviation or median and interquartile range [Q1–Q3] according to their distribution (respectively normal or not).

Graphically, study results were presented either in bar graphs, lines of repeat measures or boxplots. The nature of the relationship between two quantitative variables of interest was assessed using non‐parametric regression via smoothing splines quantitatively expressed as a linear regression with the *R* ^2^ coefficient.

Quantitatively, the statistical significance of study results was assessed either using ANOVA test with multiple comparison of means, the Kruskal‐Wallis test of multiple medians, or the Student's *t*‐test of two means as appropriate. All tests were two‐sided and statistical significance was set at *p* < .05. The data were analysed using R statistical software (version 4.1.0; R Foundation for Statistical Computing, Vienna, Austria; http://www.r‐project.org).

Our quantitative results are detailed in supplementary File 1.

### 2.21 Study approval

For human samples, in compliance with French bioethics law (2004‐800; 8 June 2004), all patients had been informed of the research use of the part of their samples remaining after diagnosis had been established, and none opposed it. Informed consent was obtained from each patient.

For in vivo experiments, the National Ethics Committee for experimental animal studies approved this study (APAFIS#17190‐2018101814245111). The ethics guidelines that were followed met the standards required by the US guidelines.^20^ Animals were maintained in the pathogen‐free housing at Sorbonne Paris Nord University (agreement number: C9300801).

### 2.22 Data availability

The data generated in this study are available within the article and its supplementary data files.

## 3 RESULTS

### 3.1 PROM2 expression level has an impact on melanoma invasion and migration, but not on cell proliferation

**FIGURE 1:**
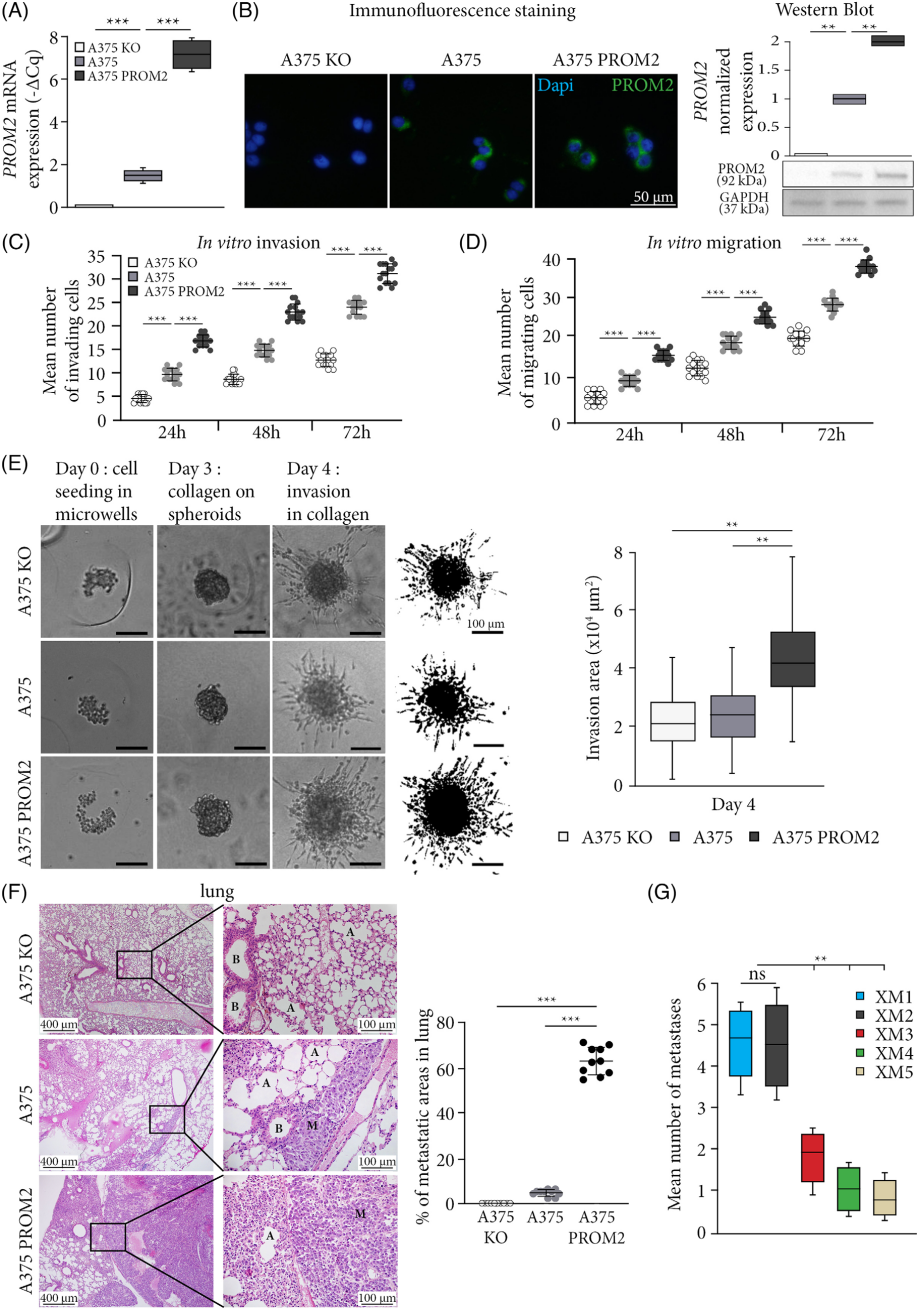
PROM2 expression level has an impact on melanoma invasion and migration. (A) mRNA expression level of *PROM2* in A375 KO (Cq > 40), A375 and A375 PROM2 cell lines (***p < .001). (B) PROM2 immunofluorescence staining (in green) with nuclei stained with DAPI (in blue), and protein normalised expression using Western blot (**p < .01). (C and D) Count of invading (C) or migrating (D) cells at 24, 48 and 72 h in Boyden chamber (***p < .001). (E) The left panel displays images illustrating the formation and expansion of spheroids derived from A375 KO, A375 and A375 PROM2 cell lines. On the right, the outcomes of the thresholding process are presented, conducted on the images acquired at day 4 after 24 h of invasion in collagen. Additionally, thresholded images for all analysed spheroids are included in Figure S4. Scale bars = 100 µm (**p < .01). (F) Left panel shows histological sections of lungs from mice injected with 5 × 10^6^ A375 KO, A375 or A375 PROM2 cell lines, using standard haematoxylin and eosin coloration (A, pulmonary alveoli; B, bronchus; M, metastasis). Right panel shows the mean surface area in percentage of lung metastases/total lung surface area quantified 8 weeks after injection (***p < .001). (G) Mean number of metastases in the five mouse models of patient‐derived melanoma xenografts (**p < .01).

Based on our results obtained in patients with metastatic melanoma,^4^ we decided to model the effects of PROM2 expression on metastatic potential, using two different types of murine models: (i) xenografts derived from two melanoma cell lines, A375 or Sk‐Mel‐28; (ii) five patient‐derived melanoma xenografts from metastasis biopsies, named XM1 to XM5. We modulated the expression of PROM2 in the two melanoma cell lines, either by transfection with a plasmid activating *PROM2* promoter or a KO plasmid. For further experiments, we selected the clone that overexpressed *PROM2* the most and the clone that did not express *PROM2* (Figures 1A and S3A). At the protein level, PROM2 expression gradually increased from the KO clone (no expression) to the native cell line and then to the overexpressing clone (Figures 1B and S3B).

We first assessed migration and invasion in Boyden chambers with or without Matrigel® coating, using these three clones for each melanoma cell line A375 and Sk‐Mel‐28. After 24 , 48 and 72 h, cell invasion and cell migration gradually increased across A375 KO, A375 and A375 PROM2 cells (Figures 1C and D). Similar results were obtained with the Sk‐Mel‐28 cell line (Figures S3C and S3D). Furthermore, we assessed the migration potential of spheroids obtained from melanoma cell lines. To that purpose, the three clones of the A375 cell line (A375 KO, A375 and A375 PROM2) were seeded in microwells to form spheroids over 3 days, and collagen was poured on top of each microwell to trigger invasion. Two days later (day 5), the migration area of A375 PROM2 spheroids was significantly larger than for A375 and A375 KO spheroids (Figures 1E and S4).

We then grafted nude mice subcutaneously with 5 × 10^6^ melanoma cells of each clone. They were euthanised on average 40 days after grafting on the basis of the size of the tumour, but there was no distant metastasis with any of the clones at this stage of development. In contrast, using the A375 cell line, 5 × 10^6^ cells injected intravenously in nude mice induced lung metastases 8 weeks after injection, although limited in numbers. *PROM2* overexpression (A375 PROM2 clone) dramatically increased the metastatic potential of the A375 melanoma cell line with massive lung and pleural metastases, and also heart and muscle metastases (Figure S3E). The mean surface area of the lung and pleural metastases was 61 ± 7% for mice injected with the A375 PROM2 cell line compared with 4 ± 2% (*p* < .01) for the A375 native cell line (Figure 1F). Similar results were found for mice injected with Sk‐Mel‐28 and Sk‐Mel‐28 PROM2 cell lines (Figure S3F). For KO cell lines, we never observed any metastasis.

In the five patient‐derived melanoma xenografts (PDX), we identified two models with a significantly higher PROM2 expression compared with the three other models (XM1 and XM2). We systematically analysed all organs and showed a significant threefold increase in numbers of metastases in XM1 and XM2 models (Figure 1G; *p* < .01).

Then, we wanted to assess whether the metastatic potential linked to PROM2 overexpression was related to cell proliferation. The level of PROM2 expression did not influence cell proliferation, whatever the melanoma cell line, neither in vitro, nor in vivo (Figures S5A and B). For the five patient‐derived melanoma xenografts, there was no correlation between tumour volume doubling time and the level of *PROM2* mRNA expression (Figures S5C and D).

Overall, PROM2 expression level has an impact in vitro and in vivo on melanoma invasion and migration, but not on cell proliferation. We thus wondered whether this increased invasive and migration phenotype linked to PROM2 expression was associated to an epithelial to mesenchymal phenotype.

### 3.2 PROM2 overexpression is associated with an EMT phenotype

**FIGURE 2:**
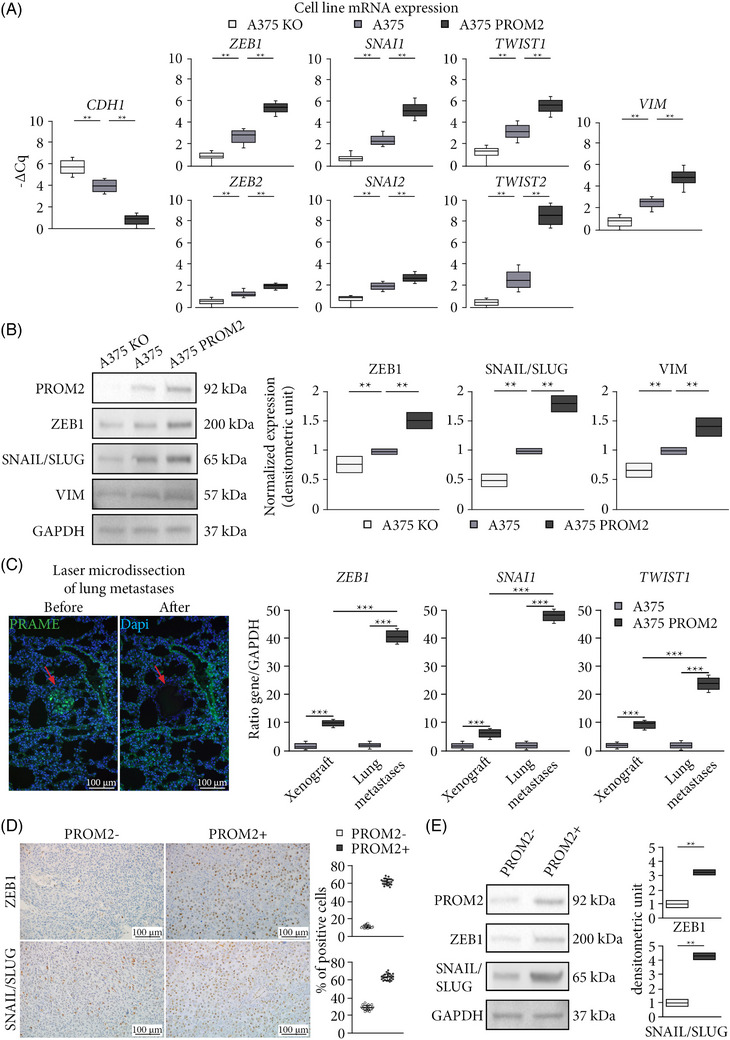
PROM2 overexpression in cells is associated with an EMT phenotype. (A) mRNA expression level of epithelial marker (*CDH1*), EMT markers (*ZEB1*, *ZEB2*, *SNAI1*, *SNAI2*, *TWIST1*, *TWIST2*) and mesenchymal marker (*VIM*) of A375 KO, A375 and A375 PROM2 cell lines (**p < .01). (B) Immunoblotting for ZEB1 and SNAI1, with normalised expression in A375 KO, A375 and A375 PROM2 cell lines (**p < .01). (C) Left panel illustrates the laser‐microdissection of a melanoma lung metastasis tagged with PRAME (green fluorescence, red arrow) from a mice injected with A375 cell line. Right panel shows mRNA quantification of *ZEB1* (***p < .0001), *SNAI1* (***p < .001) and *TWIST1* (***p < .001) in laser‐microdissected lung metastases or tumour xenografts using digital‐droplet PCR. (D) ZEB1 and SNAIL/SLUG immunostaining of tumour xenografts expressing low (PROM2‐) or high (PROM2+) PROM2 (***p < .0001). (E) PROM2, ZEB1 and SNAIL/SLUG immunoblotting of tumour xenografts expressing low (PROM2−) or high (PROM2+) PROM2 (**p < .01).

For the A375 cell line, we showed a gradual significant decrease in *CDH1* (e‐cadherin epithelial marker) mRNA expression while *VIM* (vimentin mesenchymal marker) mRNA expression significantly increased from A375 KO to A375 and to A375 PROM2. In addition, PROM2 overexpression was associated with increased mRNA expression for all six EMT markers *ZEB1*, *ZEB2*, *SNAI1*, *SLUG*, *TWIST1*, *TWIST2* (transitional markers) (Figure 2A). Western blot for ZEB1 and SNAIL/SLUG showed the same results than for mRNA (Figure 2B). A similar pattern was observed for Sk‐Mel‐28 (Figures S6A and B).

We then assessed in vivo mRNA expression of EMT markers in subcutaneous xenografts derived from cell lines and found similar results to those obtained in vitro, both for the A375 and the Sk‐Mel‐28 cell lines (Figures S7A and B). In mice injected intravenously with A375 cell line, mRNA expression of EMT markers did not increase significantly in laser‐microdissected lung metastases (PRAME‐positive cells; Figure 2C) compared with subcutaneous tumour xenografts. In contrast, in lung metastases derived from the A375 PROM2 clone, EMT‐marker expression was much higher than in subcutaneous tumours obtained with the same clone (Figure 2C).

We also assessed the EMT phenotype in melanoma PDXs. In the 78 PDXs analysed, the median (−ΔCq) for *PROM2* mRNA expression was 12. Using it as a cut‐off value, we distinguished two groups of PDXs: the PROM2+ group with a *PROM2* mRNA expression ≥ 12 and the PROM2‐ group with a *PROM2* mRNA expression < 12. *CDH1* expression was significantly lower in PROM2+ than in PROM2‐ tumours (*p *= .002) whereas other markers including *VIM* had an increased expression (*p *= .002) (Figure S4C). Using immunostaining, we also found a significantly larger number of cells expressing the EMT markers ZEB1 and SNAIL/SLUG in PROM2+ tumours (respectively 68 ± 5 and 82 ± 6%) compared with PROM2‐ tumours (respectively 7 ± 6 and 11 ± 4%, *p *< .01 for the two markers) (Figure 2D). Western blot for ZEB1 and SNAIL/SLUG on tumours confirmed these results (Figure 2E).

Overall, the invasive and migration phenotypes linked to PROM2 overexpression, and thus the increased metastatic potential, are associated with an EMT phenotype in melanoma models. We then intended to demonstrate the link between PROM2 expression and a second cancer hallmark that is resistance to cell death.

### 3.3 PROM2 overexpression is associated with ferroptosis resistance

**FIGURE 3:**
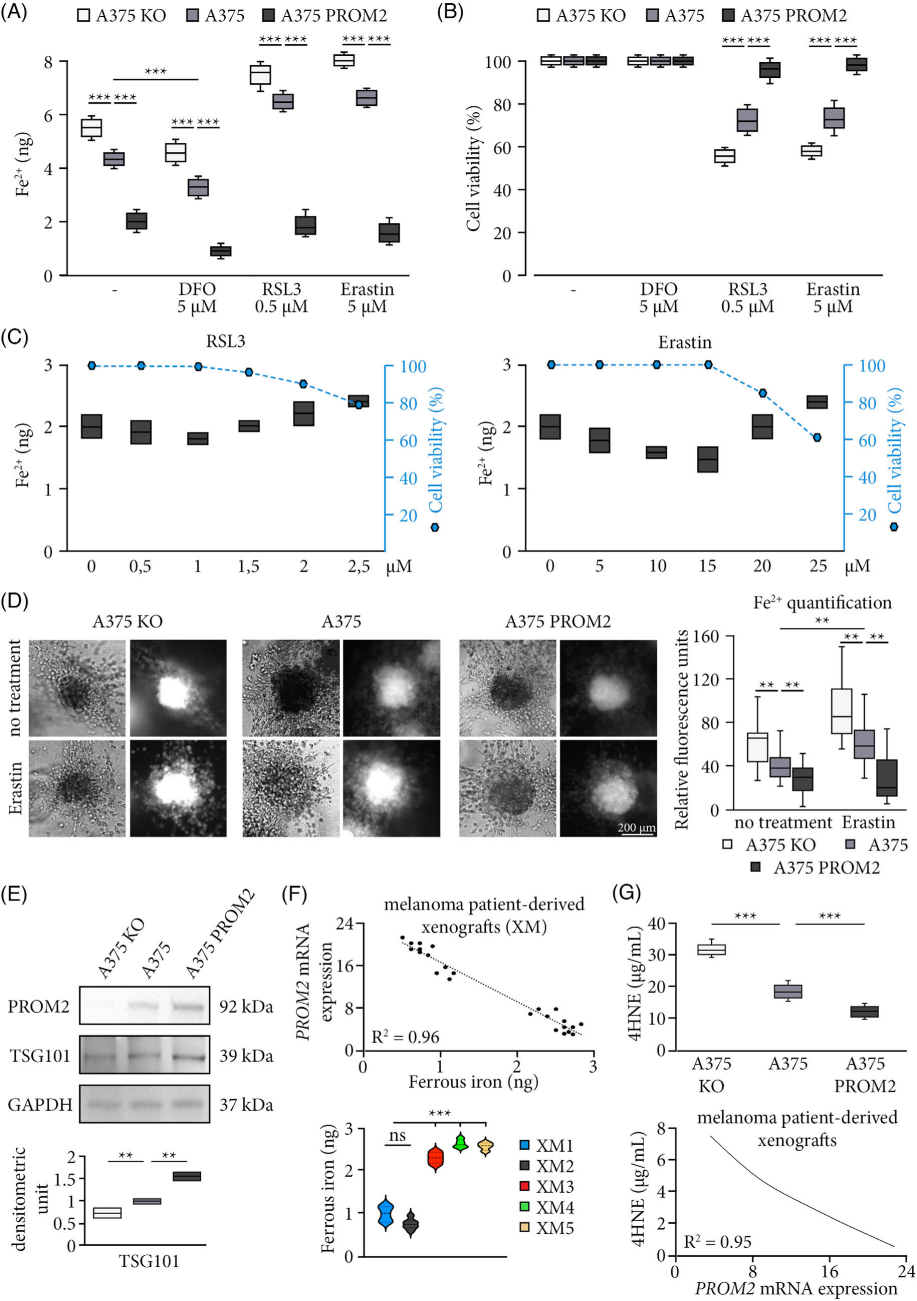
PROM2 overexpression is associated with ferroptosis resistance. (A) Quantification of ferrous iron (Fe^2+^) in A375 KO, A375 and A375 PROM2 cell lines without treatment, or after 24 h of treatment with deferoxamine (DFO, ferroptosis inhibitor), or with RSL3 or Erastin (ferroptosis activators) (***p < .0001). (B) Cell viability of A375 KO, A375 and A375 PROM2 cell lines after the addition of DFO, RSL3 or Erastin during 24 h (***p < .0001). (C) Quantification of Fe^2+^ (bar histograms) and cell viability (dash‐dotted line) of A375 PROM2 cell line treated for 24 h with increasing concentrations of RSL3 or Erastin. (D) Left panel shows spheroids obtained from A375 cell lines and cultured with or without Erastin [using white light (left) or FerroOrange fluorescence (right)]. Right panel shows Fe^2+^ quantification through FerroOrange fluorescence quantification (**p < .01). (E) Immunoblotting and quantification of TSG101 (multivesicular bodies) in A375 KO (white), A375 (grey) and A375 PROM2 (black) cell lines (**p < .01). (F) Quantification of Fe^2+^ in patient‐derived melanoma xenografts, with the correlation with *PROM2* mRNA expression in tumours (***p < .0001). (G) Quantification of 4HNE (lipid peroxidation) in A375 KO, A375 and A375 PROM2 cell lines (top panel, ***p < .0001), and the correlation with *PROM2* mRNA expression in patient‐derived melanoma xenografts (bottom panel, ***p < .001).

Since PROM2 may be linked to ferroptosis resistance in vitro^9^ with ferrous iron (Fe^2+^) cytoplasmic accumulation and cell death,^10^ we wanted to assess this process in our in vivo metastatic melanoma models. In melanoma cells, we found a significant decrease in the amount of Fe^2+^ when *PROM*2 expression increased (Figure 3A). When we added DFO, a ferroptosis inhibitor, the level of Fe^2+^ decreased significantly in the three cell lines (*p* < .05). In contrast, when we added a ferroptosis activator, either RSL3 or erastin, the level of Fe^2+^ significantly increased for the A375 KO and A375 cell lines (*p* < .05) alongside increased cell death (Figure 3B). Surprisingly, Fe^2+^ levels did not vary for the A375 PROM2 cell line and this was associated with an absence of cell death and ferroptosis resistance. Furthermore, in A375 PROM2 cells, at least four times higher concentrations of RSL3 or erastin were needed to bypass this resistance, then inducing a significant decrease in cell viability (Figure 3C). We found similar results for the Sk‐Mel‐28 cell line (Figures S8A–C).

We then wanted to assess if this link between *PROM2* overexpression and resistance to ferroptosis was also true in a three‐dimensional model. The A375 melanoma clones were seeded in microwells to form spheroids invading collagen. The level of Fe^2+^ also significantly decreased when *PROM*2 expression increased (Figure 3D). The addition of erastin to the spheroids significantly increased the amount of Fe^2+^ for the A375 KO and A375 cell lines, but, once again, did not impact the quantity detected for the A375 PROM2 cell line (Figure 3D).

Quantifying TSG101 protein level, a marker of microvesicular bodies containing ferrous iron, we showed that it significantly increased with PROM2 expression (Figure 3E), suggesting a link between the numbers of microvesicular bodies and PROM2 expression. Since microvesicular bodies are a major source of exosomes, exosomes were isolated from A375 and A375 PROM2 by differential ultracentrifugation. Using NTA, the size distribution ranged from 50 to 200 nm in diameter (Figure S9A). A375 PROM2 cells were found to generate more abundant exosomes than the A375 cells (Figure S9B). No difference in the mean size of exosomes derived from both cell lines was detected (Figure S9C). Using f‐NTA to determine the relative percentage of tetraspanin subpopulations in the exosomal fractions, no significant difference was observed between exosomes derived from the two cell lines (Figure S9D). Electron microscopic imaging displayed the presence of heterogenous population of exosomes of different sizes (Figure S9E). All vesicles showed round or cup‐shaped morphology surrounded by a double or single membrane. Some less abundant large vesicles in the size range of 200 nm were displayed high dense granulated cytoplasmic materials, whereas the smaller vesicles, which represent the highest population possessed less dense cytoplasmic content. Western blotting revealed the presence of positive exosome markers including Alix, CD9, CD81, Flotillin‐1 and TSG101 (Figure S9F). The purity of the isolated exosomes was confirmed by the absence of the cellular endoplasmic reticulum protein Grp94 (negative exosome marker). β‐Actin was used as a loading control.

In parallel, we quantified Fe^2+^ in the five patient‐derived melanoma xenografts (total of 25 tumours) and found a significantly lower level of Fe^2+^ in XM1 and XM2 compared with the other three models, inversely correlated with *PROM2* mRNA expression levels (Figure 3F).

Lipid peroxidation, which is a final step of ferroptosis, was assessed by 4‐HNE levels, showing a significant decrease when *PROM2* expression increased. In the five patient‐derived melanoma xenografts, a negative correlation was also found between *PROM2* mRNA expression and lipid peroxidation (Figure 3G).

Overall, our results demonstrated that *PROM2* overexpression was directly associated with ferroptosis resistance in human melanoma both in vitro and in vivo. We thus confirmed our hypothesis that in human metastatic melanoma PROM2 appears to be the cornerstone of two cancer hallmarks: the metastatic process and resistance to ferroptotic cell death. We wondered whether this could be observed in other cancer types.

### 3.4 Metastatic potential linked to PROM2 overexpression is not specific to melanoma cancer type

We then wondered if the metastatic potential associated with PROM2 overexpression was specific or not to melanoma, as suggested for kidney, lung, pancreas and ovarian cancers^29^, ^30^, ^31^ (Table S1). Using PDXs of human renal cell carcinomas and triple negative breast cancers, we assessed *PROM2* and EMT marker mRNA expression. We found a correlation between *PROM2* expression and the three EMT markers *ZEB1*, *SNAI1* and *TWIST1* in both cancer types (Figure S10A). Again, we assessed the link between *PROM2* expression and ferroptosis resistance in PDX models of renal and breast cancers. We found an inverse correlation between tumour *PROM2* expression and ferrous iron levels and also between *PROM2* expression and lipid peroxidation, in both cancer types (Figure S10B).

### 3.5 Targeting PROM2 with an ASO prevents metastases in human melanoma xenografts

**FIGURE 4:**
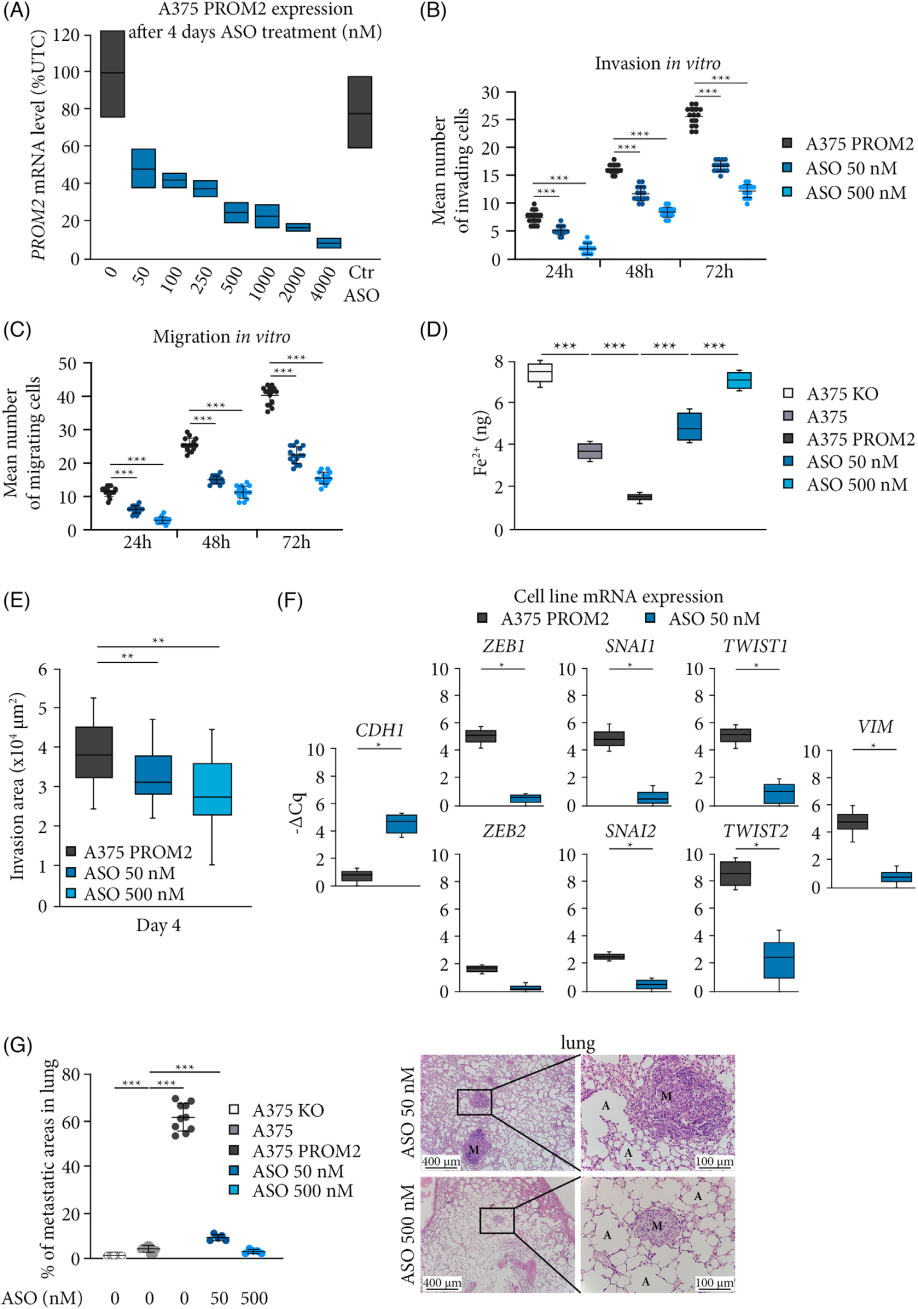
Targeting PROM2 with an antisense oligonucleotide (ASO) prevents metastases in human melanoma xenografts. (A) *PROM2* mRNA expression of A375 PROM2 cells treated during 4 days with different concentrations of an anti‐PROM2. (B) Counts of invading A375 PROM2 cells treated for 72 h with two concentrations of the ASO (***p < .0001). (C) Counts of migrating A375 PROM2 cells treated for 72 h with two concentrations of the ASO (***p < .0001). (D) Quantification of Fe^2+^ of A375 PROM2 cell line treated for 4 days with two concentrations of the ASO (***p < .0001). (E) mRNA expression level of epithelial marker (*CDH1*), EMT markers (*ZEB1*, *ZEB2*, *SNAI1*, *SNAI2*, *TWIST1*, *TWIST2*) and mesenchymal marker (*VIM*) of A375 PROM2 cell line treated for 4 days with 0 or 50 nM of the ASO (*p = .04). (F) Mean surface area of lung metastases determined 8 weeks after injection of 5 × 10^6^ A375 PROM2 cell line, untreated or treated in vitro for 4 days with 50 or 500 nM of the ASO before injection (***p < .001).

For therapeutic purposes, we decided to engineer an anti‐PROM2 ASO to target PROM2 that is preferentially located in intracytoplasmic microvesicular bodies. This ASO was purposely designed for gymnotic delivery. We showed that the treatment of the A375 PROM2 clone with the ASO for 4 days significantly decreased mRNA *PROM2* levels, even with very low 50 nM concentrations (Figure 4A). At 500 nM, this was associated with no PROM2 protein expression (data not shown). We then assessed the in vitro effects of ASO on cell invasion and migration. The use of the ASO at 50 and 500 nM gradually decreased invasion and migration (Figures 4B, C and S11). Alongside, the amount of Fe^2+^ in ASO‐treated cells significantly increased (Figure 4D). Using spheroid assays and the ASO at two concentrations, namely 50 and 500 nM, the migration area of A375 PROM2 spheroids was significantly decreased when the cells were exposed to the ASO at 50 and 500 nM (Figure 4E).

When we assessed the effect of anti‐PROM2 ASO on the EMT phenotype of the A375 PROM2 cell line, we also found a decreased expression of EMT markers compared with A375 PROM2 untreated cells (Figure 4F).

When we blocked PROM2 expression before intravenous injection of A375 PROM2 cells previously treated for 4 days with the ASO, the metastatic potential dramatically decreased (Figure 4G).

Overall, these results showed that use of anti‐PROM2 ASO is sufficient to dramatically decrease metastatic potential and restore ferroptosis sensitivity, mimicking the A375 KO cell line. Since PROM2 appears to be the cornerstone of two cancer hallmarks, we wondered whether the combination of these two hallmarks, by way on an aggravation loop, contributes to increasing the metastatic burden.

### 3.6 PROM2 is implicated in a pathophysiological loop of increased metastatic potential

**FIGURE 5:**
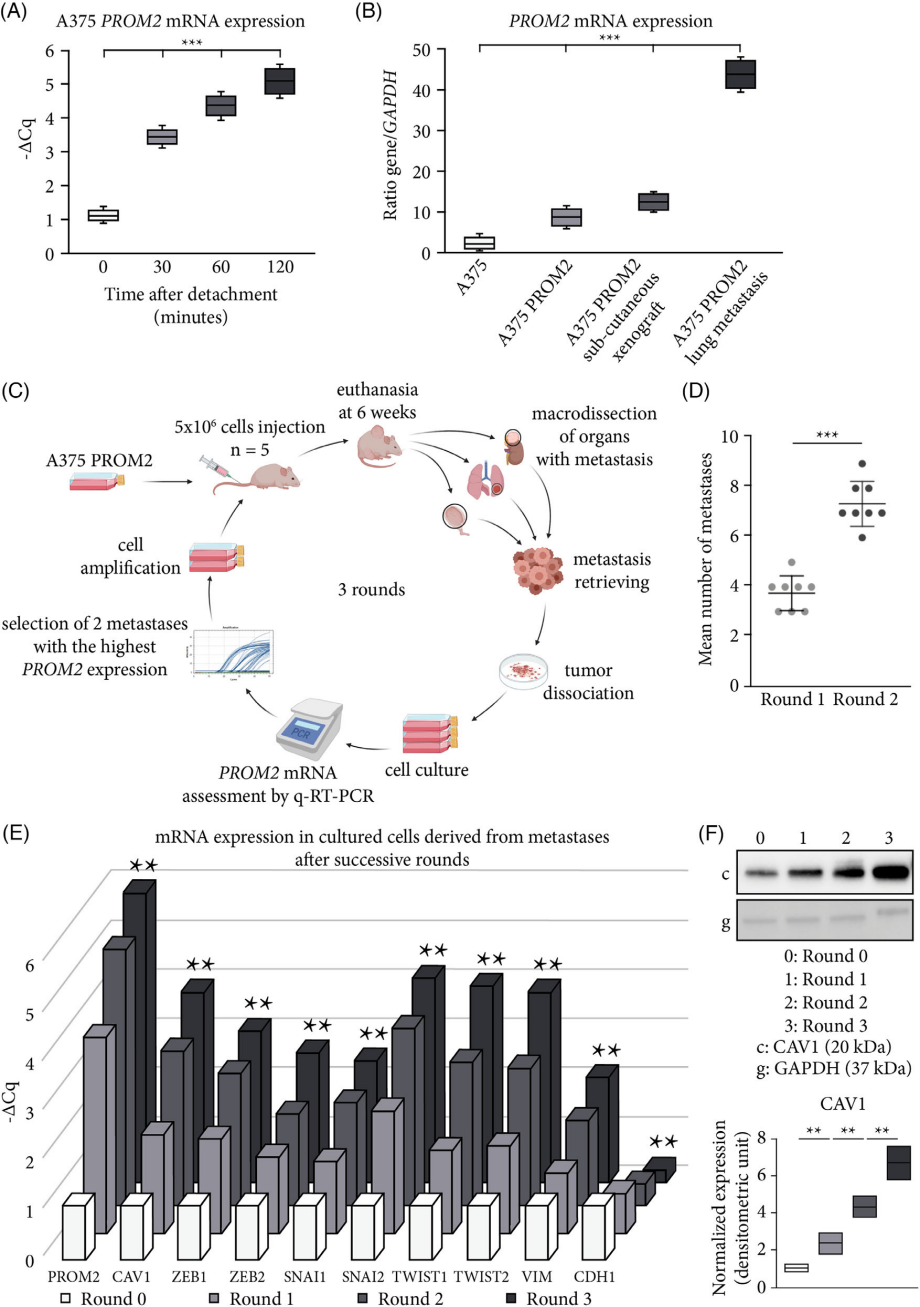
PROM2 is implicated in a pathophysiological loop of increased metastatic potential. (A) *PROM2* mRNA expression after detachment of A375 cell line for 0, 30, 60 and 120 min (***p < .0001). (B) PROM2 *mRNA* expression in A375 and A375 PROM2 cell lines, and in subcutaneous xenografts and lung metastases derived from A375 PROM2 cell line and obtained 8 weeks after grafting (***p < .0001). (C) Schematic of experimental enrichment loop (BioRender and SMART Servier Medical Art). (D) Mean number of metastases between round 1 and round 2 (***p < .001). (E) *PROM2*, *CAV1* and EMT marker mRNA expression in cultured cells after successive rounds (**p < .01 from round 0 to 3).

Since *PROM2* mRNA expression gradually increased after time lapses ranging from 30 to 120 min from cell detachment in vitro, and in lung metastases derived from the A375 PROM2 clone as compared with the native clone (Figures 5A and B), we decided to build a new model to study the metastatic disease and mimic the runaway metastatic process frequently observed in patients.

We injected intravenously a series of nude mice with A375 PROM2 cells (round 0). After 6 weeks, lung metastases (or other metastatic localisations) were macrodissected, and metastatic cells were cultured in normal medium after dissociation. After a week of in vitro amplification, cells were reinjected into a new series of nude mice (round 1). This enrichment pattern was repeated for three rounds (Figure 5C). We observed a significant increase in the numbers of metastatic localisations between round 1 and round 2 (micro and macro‐metastases; Figure 5D), thus validating our model. This amplification loop was that effective that, for round 3, we had to euthanatise mice as soon as 4 weeks after injection because of clinical signs of animal suffering and unexpected metastatic localisations. *PROM2* expression gradually increased in the cell lines and in corresponding metastases from round 0 to round 3 (Figure 5E). Since PROM2 induces caveolin‐1 (CAV1) expression and phosphorylation,^7^, ^8^ which can itself increase mRNA expression of *SLUG*, *TWIST* and *VIM*,^32^, ^33^, ^34^ we assessed the expression of all these markers across successive rounds in cell lines and derived metastases. Alongside increased *PROM2* expression, we showed a gradual but significant increase in mRNA expression of *CAV1* and EMT markers from rounds 0 to 3 (Figure 5E). We confirmed this significant increase of CAV1 expression at protein level using Western blot (Figure 5F).

### 3.7 PROM2 pathophysiological loop is linked to metastatic progression in patients

**FIGURE 6:**
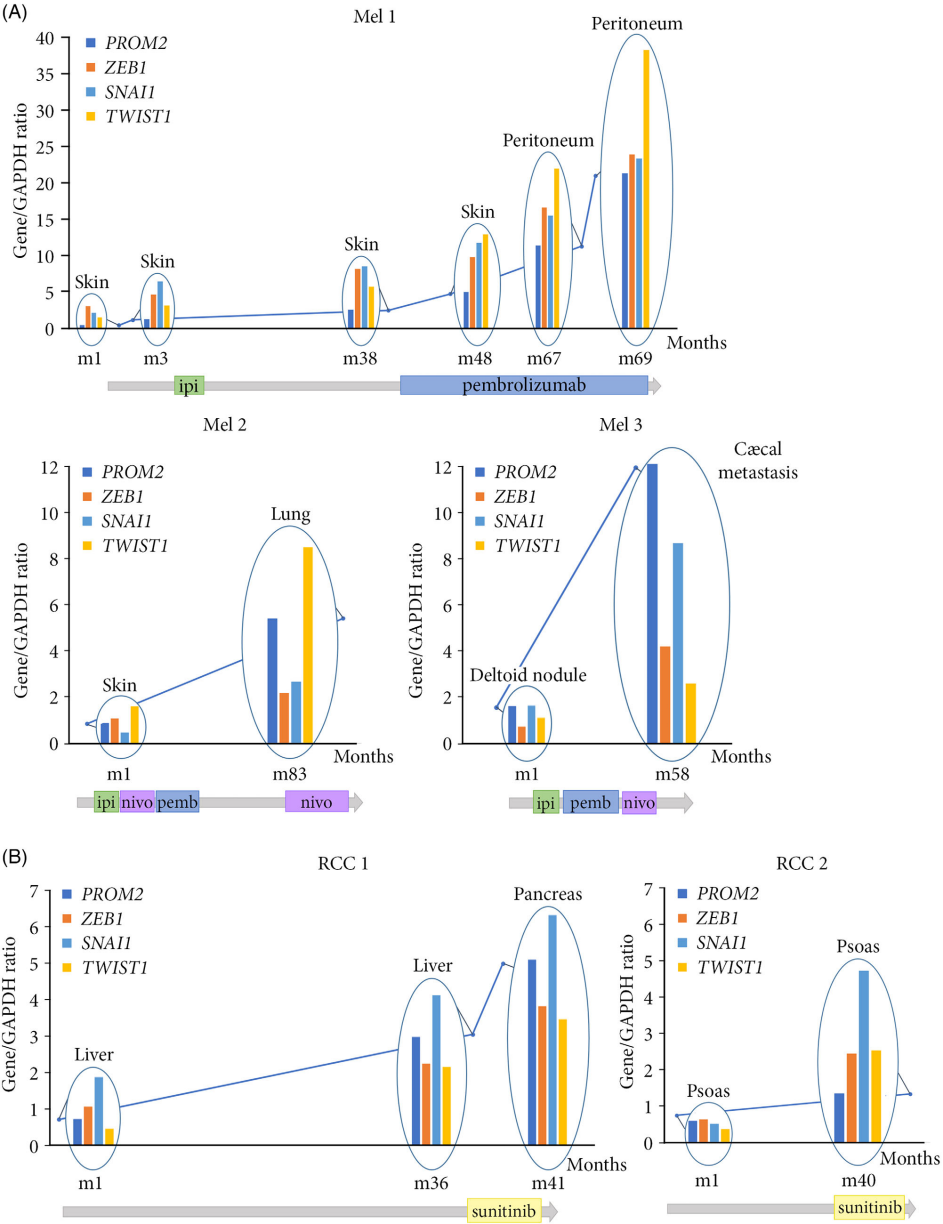
PROM2 pathophysiological loop is linked to metastatic progression in patients. (A) *PROM2* and EMT marker mRNA expression in sequential biopsies of three patients with metastatic melanoma (Patient 1: Mel1, Patient 2: Mel2, Patient 3: Mel3). (B) *PROM2* and EMT marker mRNA expression in sequential biopsies of two patients with renal cell carcinoma (Patient 1: RCC1, Patient 2: RCC2). Ipi, ipilimumab; nivo, nivolumab; pembro, pembrolizumab.

We wondered if this pathophysiological loop was also found in patients with metastatic melanoma or renal cancer. We had the opportunity to analyse exceptional sequential metastasis biopsies from melanoma patients. Using laser‐microdissection to select cancer cells, combined with ddPCR, we found that *PROM2* and EMT marker expression gradually increased with the metastatic progression in these three patients (Figure 6A). In two other patients with sequential metastasis biopsies of clear‐cell renal cell carcinoma, we obtained the same remarkable result (Figure 6B). Overall, for patients with multiple samples, we observed exponential aspect curves of the markers related to the progression of metastatic disease and leading to the patients’ death.

## 4 DISCUSSION

In this study, we demonstrated that the runaway metastatic process is closely linked to PROM2 overexpression, making PROM2 a promising bio‐target in resort situations in metastatic melanoma, and also possibly in other cancer types.

In a clinical study using biopsy samples from 101 patients with melanoma lymph node metastases, we demonstrated that high PROM2 expression was associated with a risk of distant lung and brain metastasis and shorter survival.^4^ High *PROM2* mRNA expression has also been associated with shorter survival among patients with metastatic pancreas, renal, ovarian and lung cancers.^29^, ^30^, ^31^ Here, for the first time, using melanoma cell lines injected intravenously in mice and patient‐derived melanoma xenografts, we demonstrated that the metastatic burden was linked to PROM2 expression via an EMT phenotype. We also directly demonstrated that PROM2 expression was associated with ferroptosis resistance in vivo. In vitro, PROM2 overexpression has been shown to lead to ferroptosis resistance in breast cancer cell lines.^9^ In our study, we used various relevant models to demonstrate this association in vivo. Indeed, we used patient‐derived melanoma xenografts that efficiently reflected tumour heterogeneity in human metastatic cancers^35^ but also build a new murine model to study the aggravation loop of the metastatic disease itself. We confirmed this in five patients with available sequential biopsies of metastases. Despite the fact that the number of patients is limited, sequential biopsies of metastases for the same patient are quite exceptional, because it is not usual to re‐biopsy different metastatic localisations overtime. Above all, the results are fully concordant with the aggravation loop data obtained in mice and provide a biological explanation for the phenomenon of metastatic runaway frequently observed in most patients with advanced cancers, which is of considerable added value for our study.

In human metastatic melanoma, PROM2 thus appears to be the cornerstone of two cancer hallmarks^13^, ^14^, ^15^: the metastatic process and resistance to cell death, including resistance to targeted and immuno‐therapies.^36^ The combination of these two hallmarks, by way on an aggravation loop, contributes to increasing the metastatic burden. The mechanism by which EMT marker expression is upregulated remains uncertain, but it seems closely linked to PROM2 expression modulation as shown using anti‐PROM2 ASOs.

**FIGURE 7:**
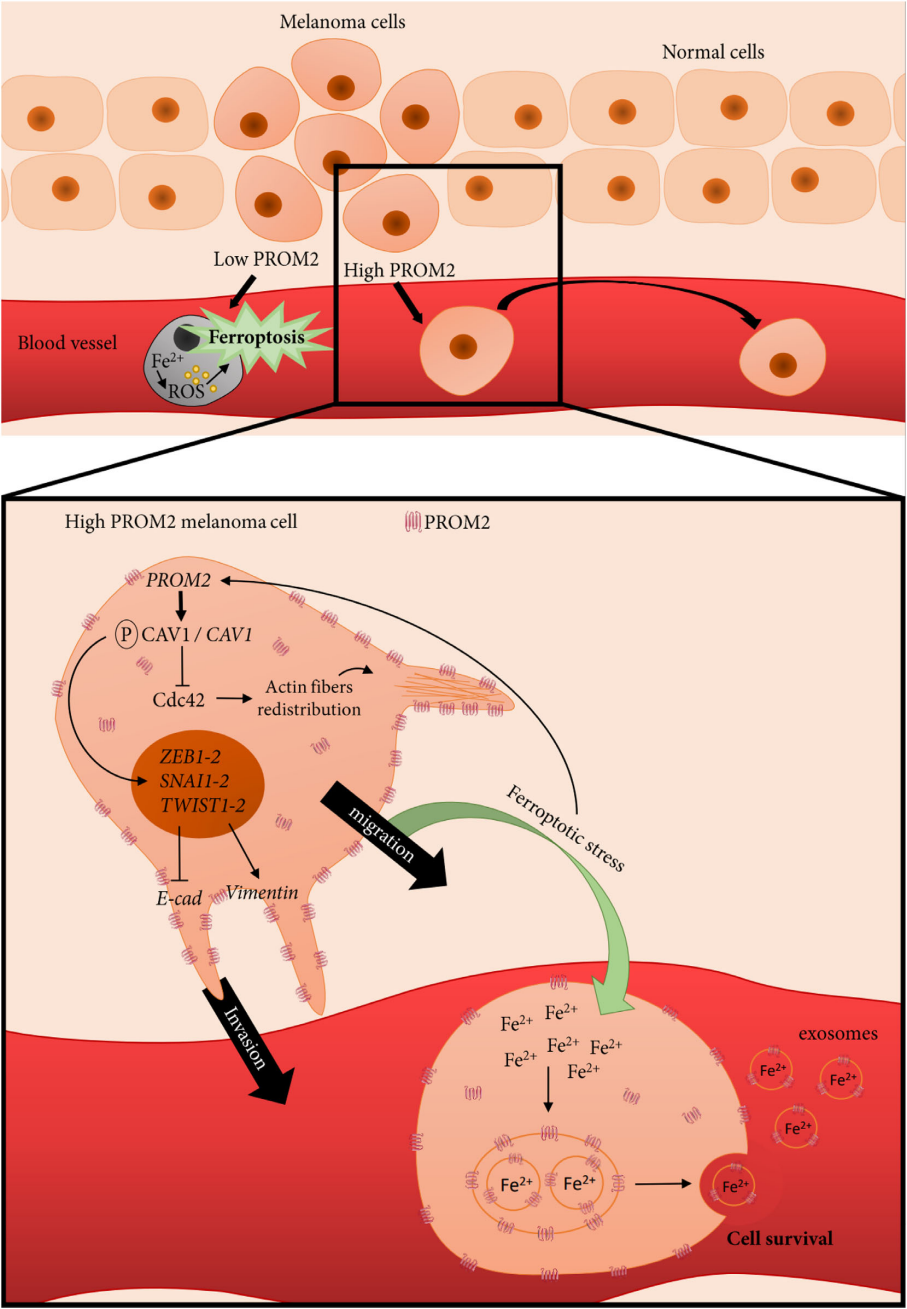
Pathophysiological loop by which high PROM2 expression promotes metastases and induces resistance to ferroptotic cell death. At the first step of the metastatic process, when a melanoma cell detaches from the tumour burden, the ferroptotic stress induces a ROS‐dependent cell death if PROM2 expression is low. In a melanoma cell with high PROM2 expression, PROM2 activates caveolin‐1 with an increase of EMT marker expression. This increase of EMT markers is associated with cell detachment and invasion. As caveolin‐1 is known to modify cdc‐42 activity and cell polarity through redistribution of actin fibres, we represent here PROM2 interplay with EMT markers and invadopode formation during invasion process. During migration in blood vessel, PROM2 overexpression overcomes ferroptotic cell death through the formation of microvesicular bodies and expulsion of Fe^2+^ from the cell. In addition, the ferroptotic stress increases itself PROM2 overexpression in an aggravating loop favouring the metastatic process.

Different pathways support the direct pathophysiological role of PROM2 (Figure 7). We showed that a ferroptotic stress directly increased PROM2 expression in vivo. We also showed in our models that *CAV1* mRNA expression increased with PROM2 expression. Since CAV1 mRNA and protein overexpression can induce EMT marker overexpression in various cancer cell lines,^37^, ^38^, ^39^, ^40^, ^41^ CAV1, that participates to caveolar endocytosis,^7^, ^8^ could be intermediate between PROM2 and EMT markers in our in vitro and in vivo melanoma models.

It has been reported that resistance to ferroptosis is closely related to the type of oxidised phospholipid present in the plasma membrane. In particular, monounsaturated fatty acids such as acid oleic and their ACSL3‐dependant acetylation decrease the membrane pool of protective polyunsaturated fatty acids and induce resistance to ferroptosis.^42^ In preclinical models of melanoma, this was particularly true in lymph circulation, thus favouring metastatic spread.^43^ These observations pave the way of studying monounsaturated fatty acids and PROM2 interactions at different membrane structures (plasma membrane, microvesicular bodies). PROM2 is a component of small membrane particles that are released into physiological fluids, such as saliva^44^ and urine.^45^, ^46^ As PROM2 is a component of intra‐cytoplasmic microvesicular bodies, it could contribute to cell–cell signalling via exosomes, promoting metastatic spread. Whether it is expressed or not by exosomes remains to be demonstrated in patients, opening perspectives for using PROM2 as a plasmatic prognosis biomarker.

Another major strength of our study is the fact that PROM2 overexpression may not only be associated with metastatic processes in melanoma, but also with other cancer types, including clear‐cell renal cell carcinomas and triple‐negative breast cancers. This is a major result that requires confirmation with additional series of patients and complementary preclinical studies.

Altogether, our results suggest that PROM2 is a promising therapeutic target. We chose an ASO approach for several reasons: (i) PROM2 protein expression is correlated with mRNA expression in our melanoma models; (ii) PROM2 protein overexpression is associated with the metastatic process during cell detachment and migration and is largely part of the membrane of multivesicular bodies to expulse Fe^2+^ in exosomes out of cancer cells to promote ferroptosis resistance,^9^, ^47^ (iii) finally PROM2 is a pentaspan protein and data are lacking on functional domains, making it difficult to engineer an appropriate therapeutic monoclonal antibody. ASOs are promising therapies that have developed widely this past two decades.^48^ From a translational perspective, an ASO seems more relevant than a monoclonal antibody, because it is capable of penetrating the cytoplasm of the cell and inhibiting the *PROM2* mRNA and therefore protein synthesis. However, they have some limitations: (i) their break‐down after administration, which can however be prevented by chemical modifications of the oligonucleotide or by a nanoparticle coating; (ii) their bioavailability and access to the tumour, which can be increased by using active targeting systems. For example, PRAME, which is a membrane protein highly expressed by human metastatic melanomas,^49^ could be used to engineer an antibody‐drug conjugate with an anti‐PROM2 ASO; (iii) their penetration into the cancer cell, requiring specific engineering of an ASO capable of gymnotic diffusion into the cell, as we achieved here. In addition, in this study, we obtained a very strong inhibitory potential even at very low concentrations of the anti‐PROM2 ASO we have engineered.

In conclusion, the metastatic burden is closely linked to PROM2 overexpression through the expression of EMT markers and ferroptosis resistance in a deterioration loop. Our results, providing a new model to study metastatic disease, open the way for further studies using PROM2 as a bio‐target in resort situations in human metastatic melanoma and also in other cancer types.

## AUTHOR CONTRIBUTIONS

*Conception and design*: G. B. *Development of methodology*: J. P. and G. B. *Acquisition of data*: All authors. *Analyses and interpretation of data*: J. P., F. P., G. B. and G. F. *Drafting and review*: J. P. and G. B. *Revision of the manuscript*: All authors. *Material support*: C. L., C. A., M. B., E. V. G. and G. B. *Administrative and technical*: G. B. *Study supervision*: G. B.

## CONFLICT OF INTEREST STATEMENT

The authors declare that they have no competing interests.

## FUNDING INFORMATION

This research received no external funding.

## ETHICS STATEMENT

In compliance with French Bioethics law (2004‐800; 8 June 2004), all patients had been informed of the research use of the part of their samples remaining after diagnosis had been established, and none opposed it. Informed consent was obtained from each patient.

The National Ethics Committee for experimental animal studies approved this study (APAFIS#17190‐2018101814245111).

## Supporting information

Supporting information

Supporting information

Supporting information

Supporting information

Supporting information

Supporting information

Supporting information

Supporting information

Supporting information

Supporting information

Supporting information

Supporting information

Supporting information

Supporting information

## Data Availability

All data generated or analysed during this study are included in this published article and its supplementary information files.
