# Supplementary figures and images for: PROM2 overexpression induces metastatic potential through epithelial‐to‐mesenchymal transition and ferroptosis resistance in human cancers
Source: Clin Transl Med. 2024 Mar 21;14(3):e1632. doi: 10.1002/ctm2.1632 (PMC10958126; doi:10.1002/ctm2.1632)

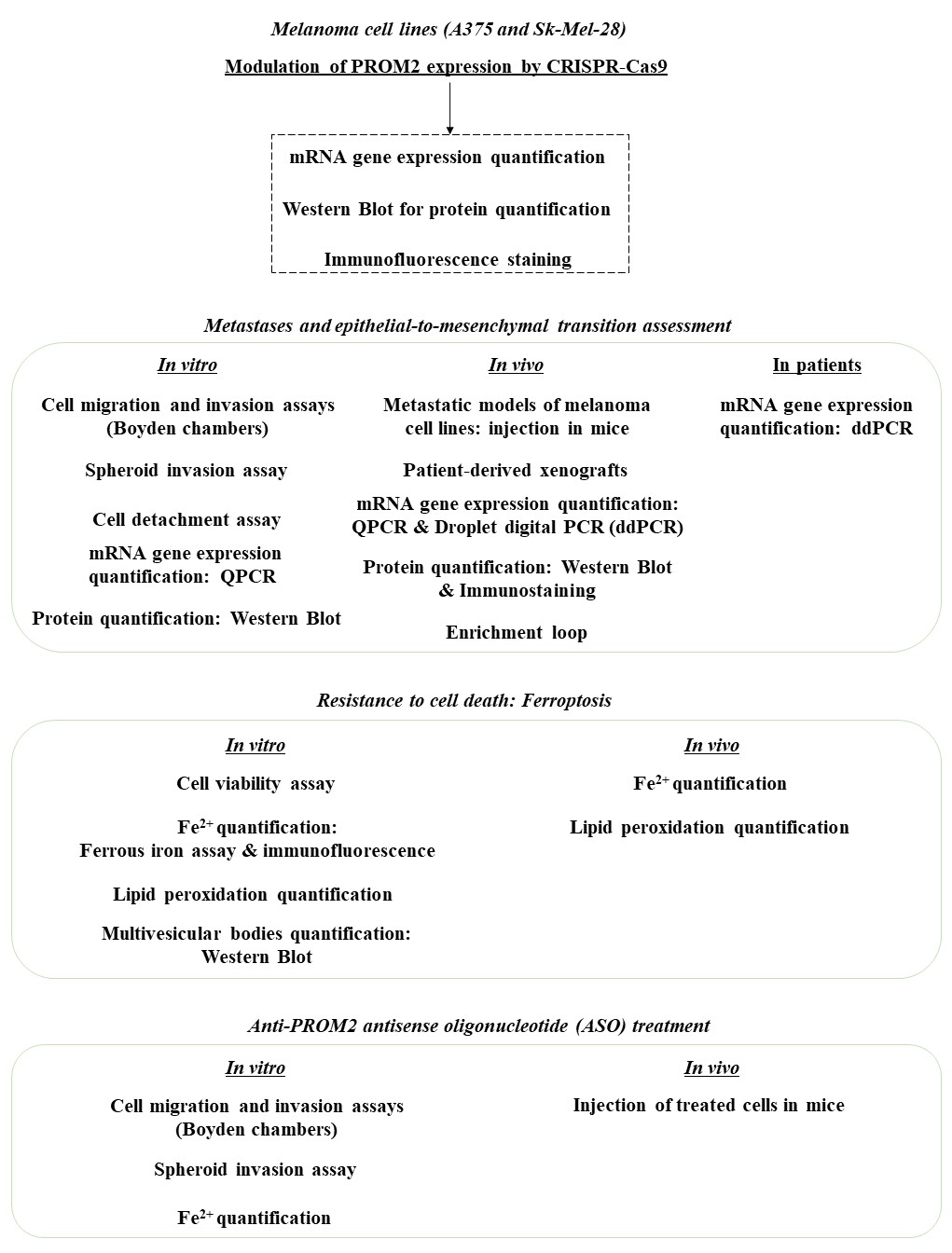

Supplement: Supplementary file 1 — Supporting information [file CTM2-14-e1632-s010.jpg]

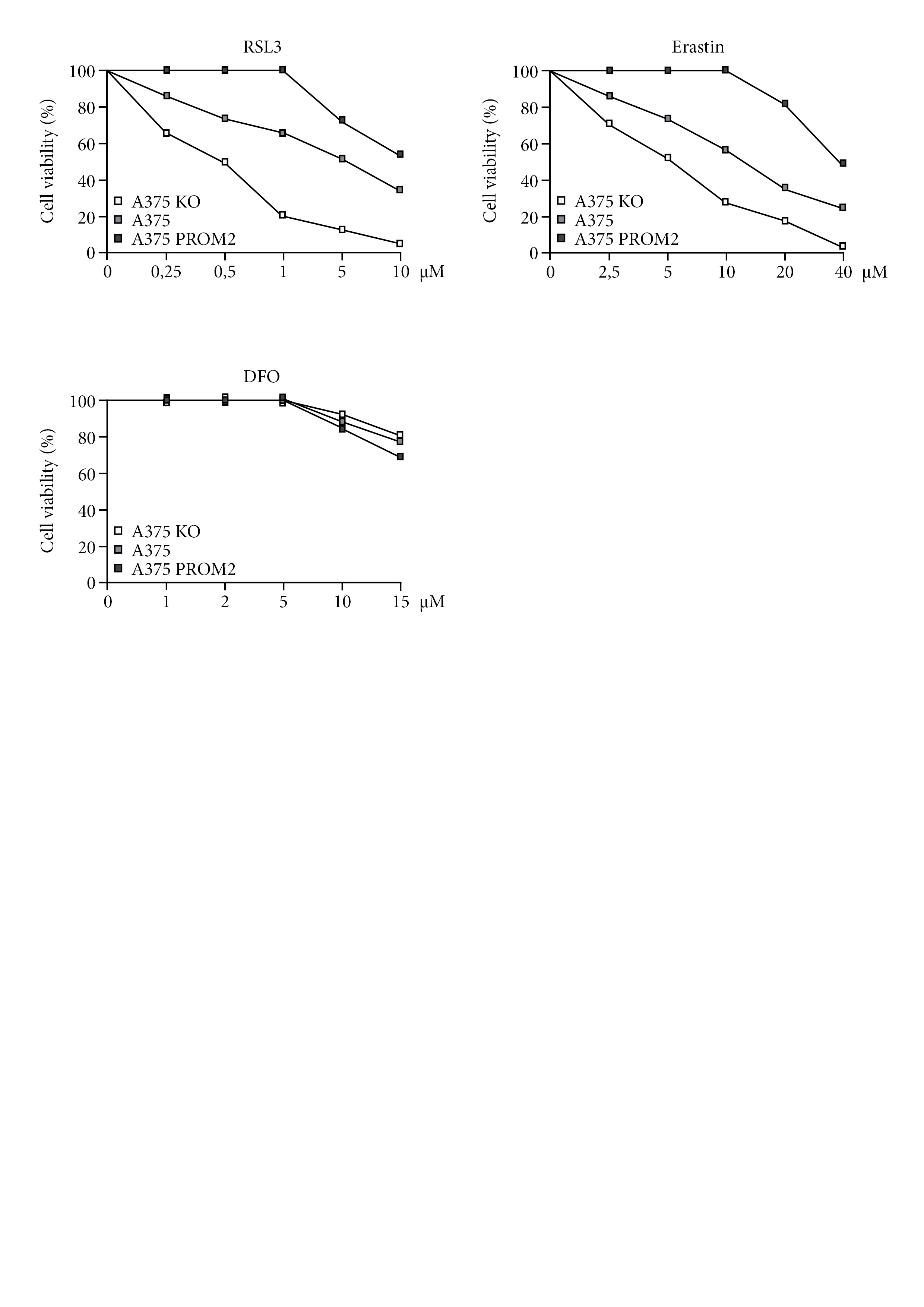

Supplement: Supplementary file 2 — Supporting information [file CTM2-14-e1632-s007.jpg]

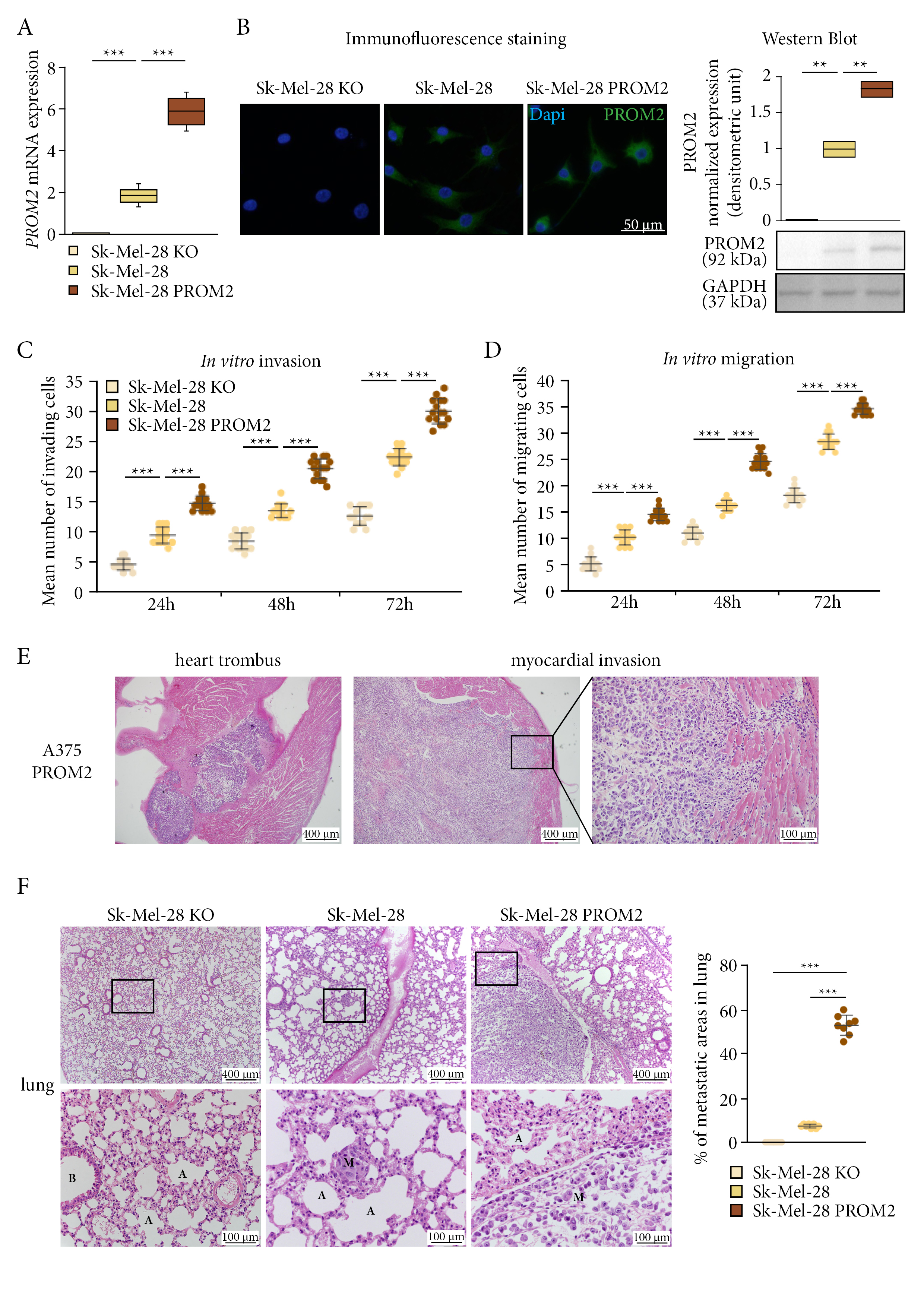

Supplement: Supplementary file 3 — Supporting information [file CTM2-14-e1632-s002.jpg]

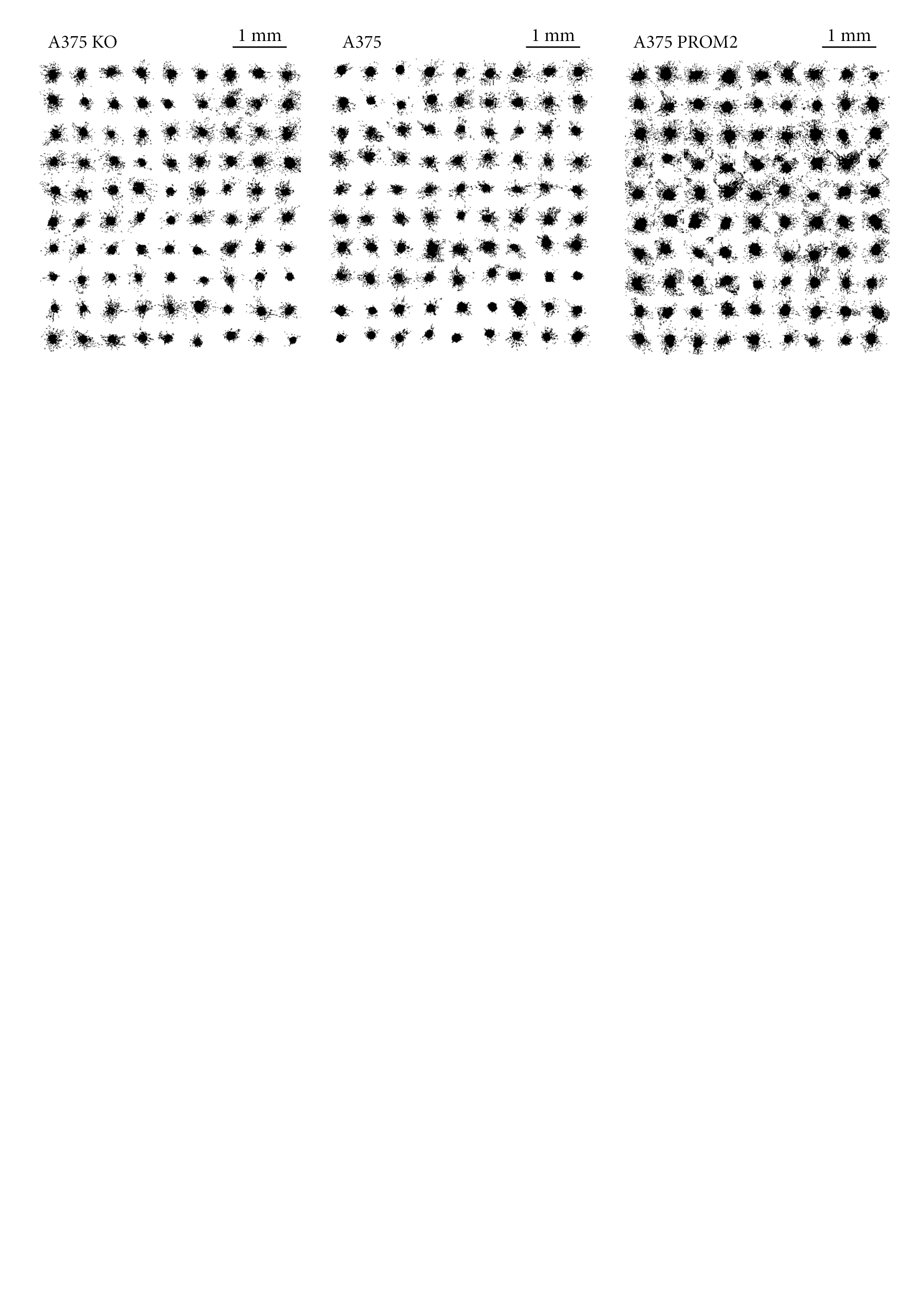

Supplement: Supplementary file 4 — Supporting information [file CTM2-14-e1632-s001.jpg]

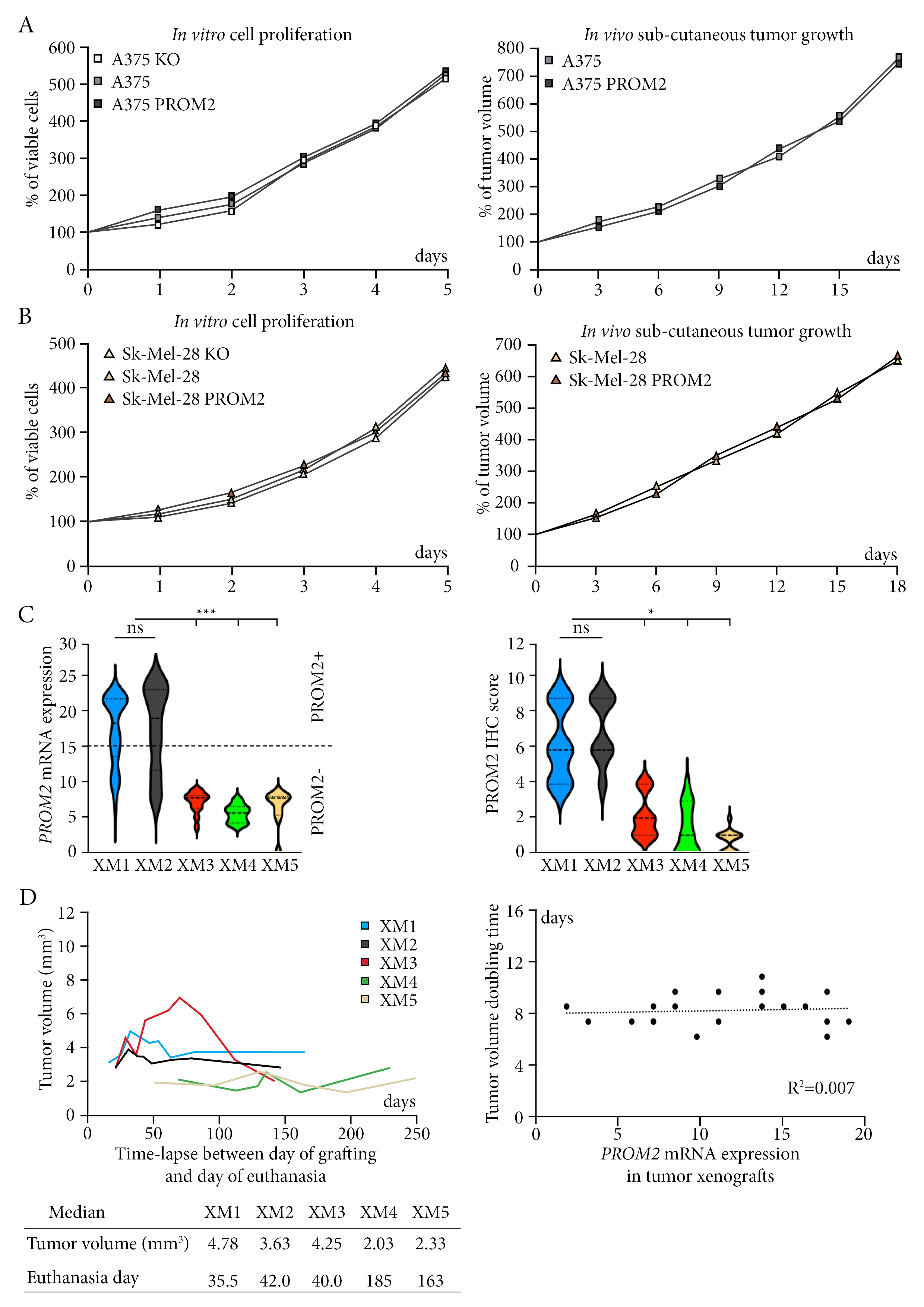

Supplement: Supplementary file 5 — Supporting information [file CTM2-14-e1632-s003.jpg]

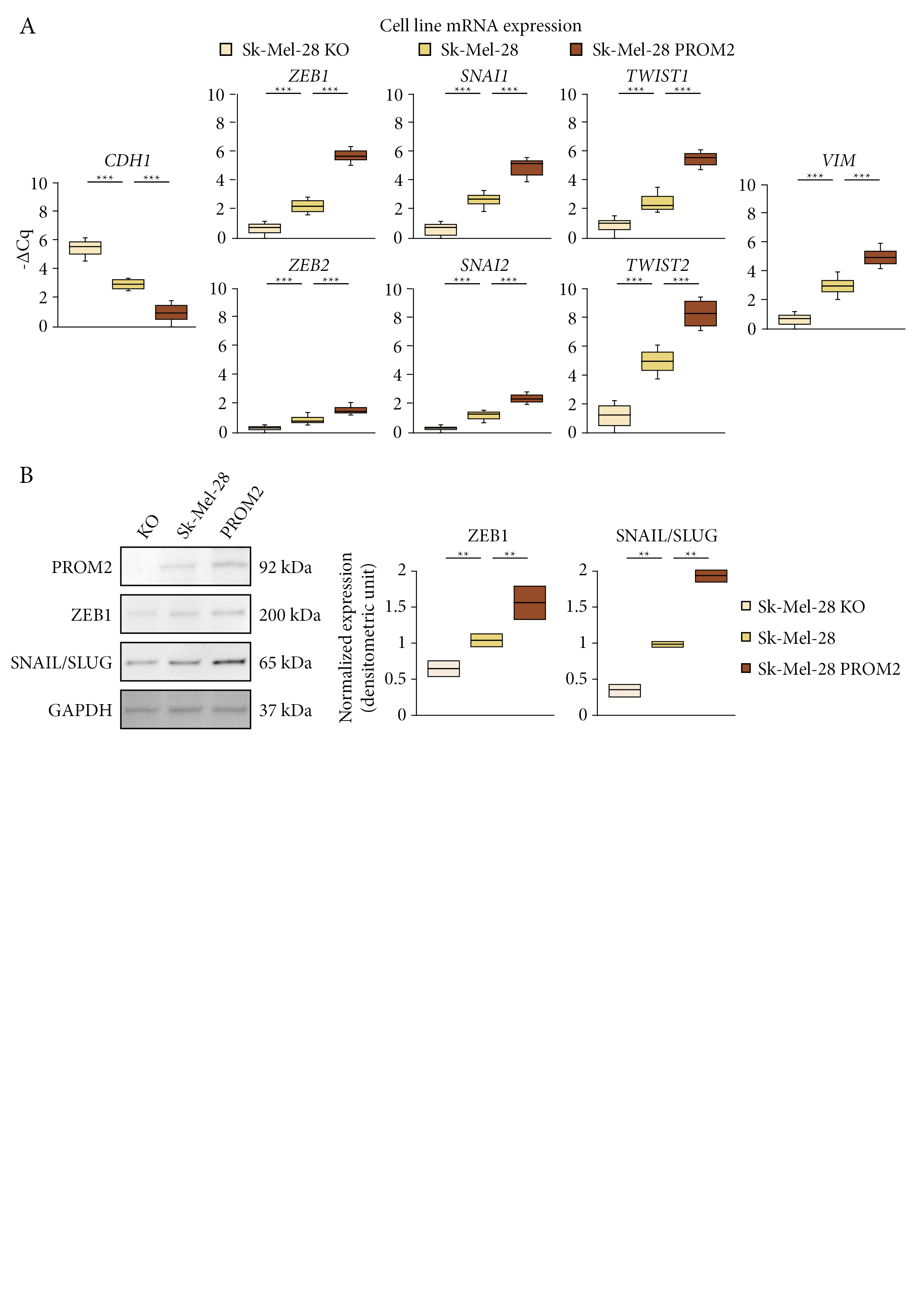

Supplement: Supplementary file 6 — Supporting information [file CTM2-14-e1632-s006.jpg]

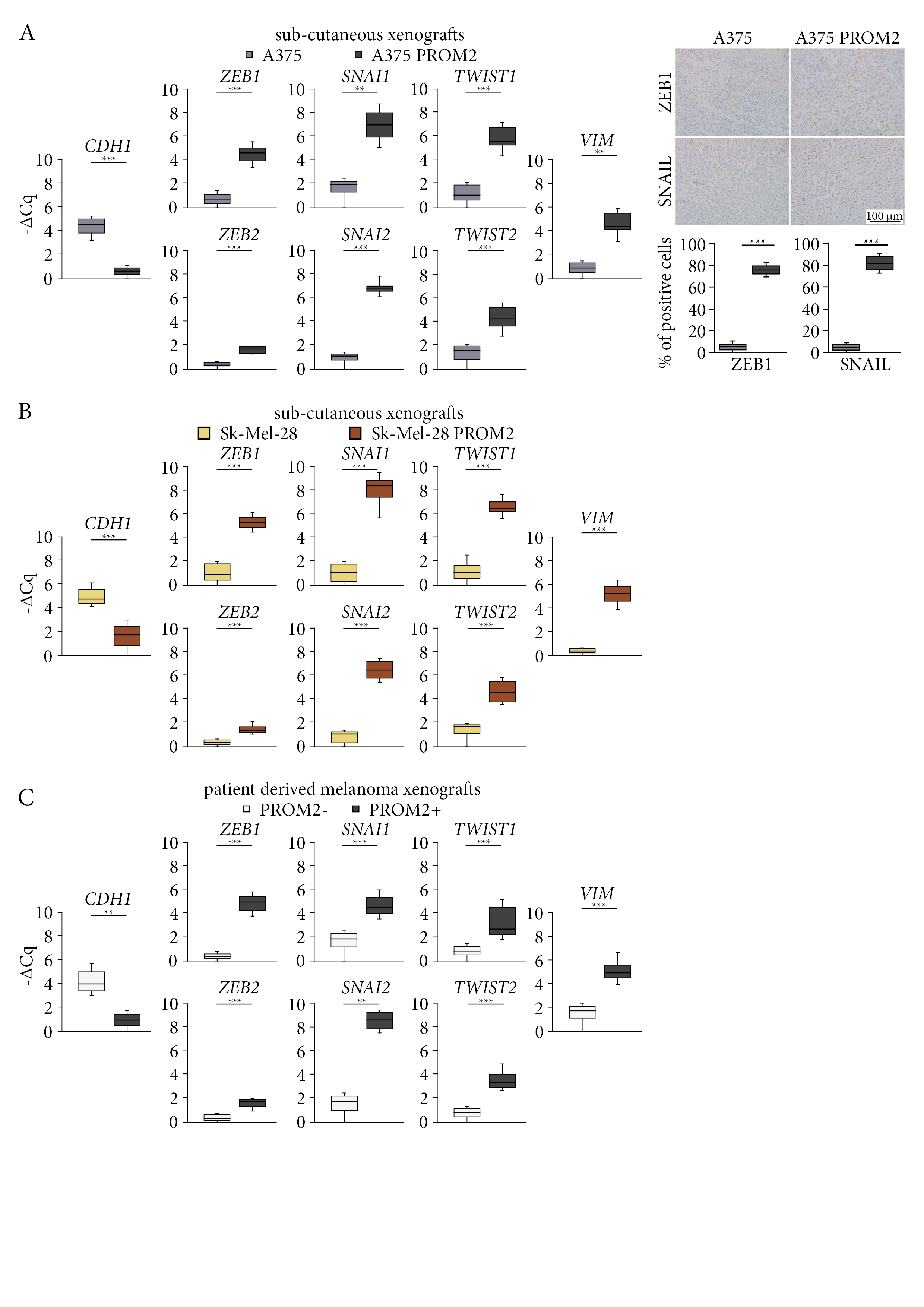

Supplement: Supplementary file 7 — Supporting information [file CTM2-14-e1632-s014.jpg]

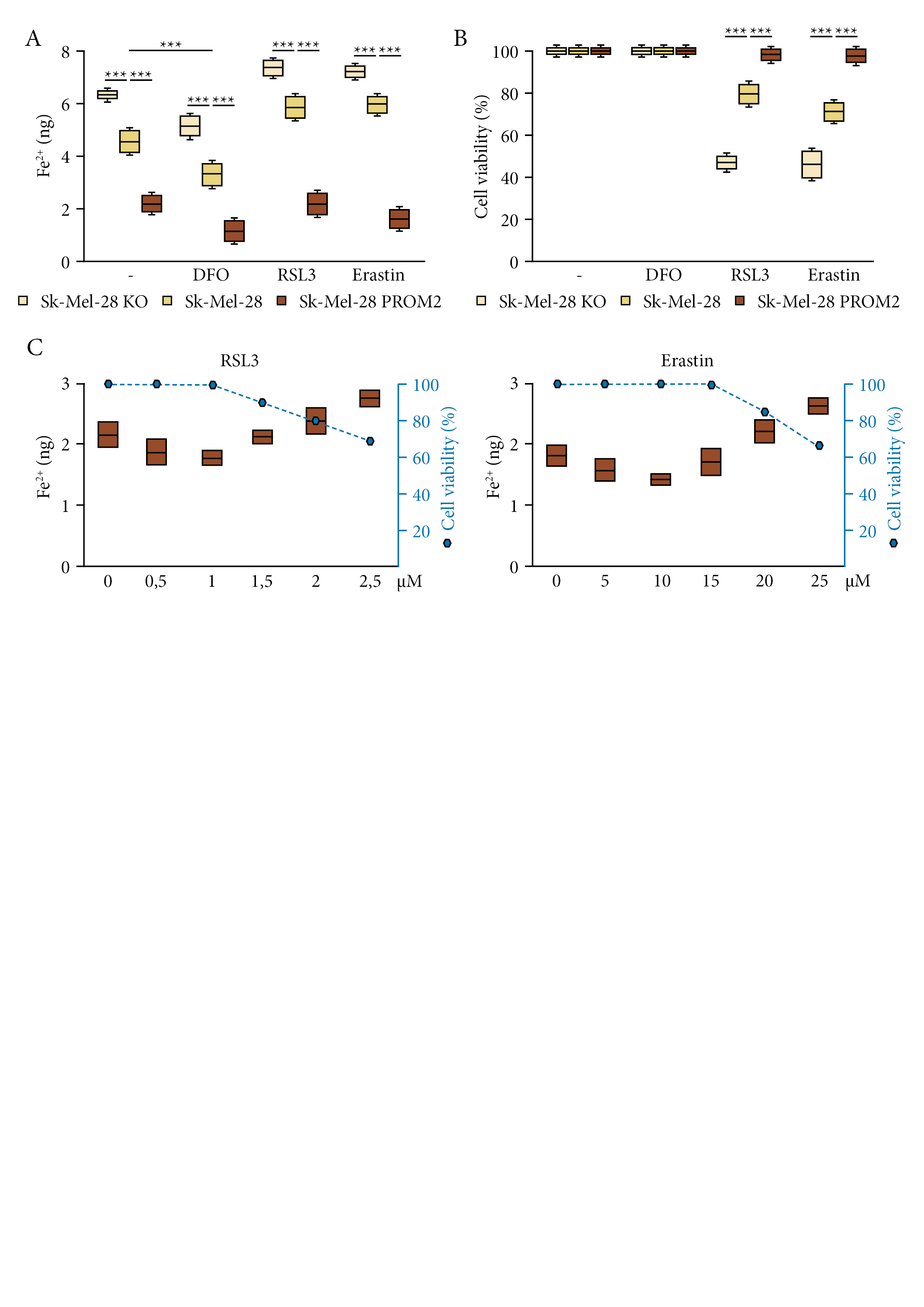

Supplement: Supplementary file 8 — Supporting information [file CTM2-14-e1632-s011.jpg]

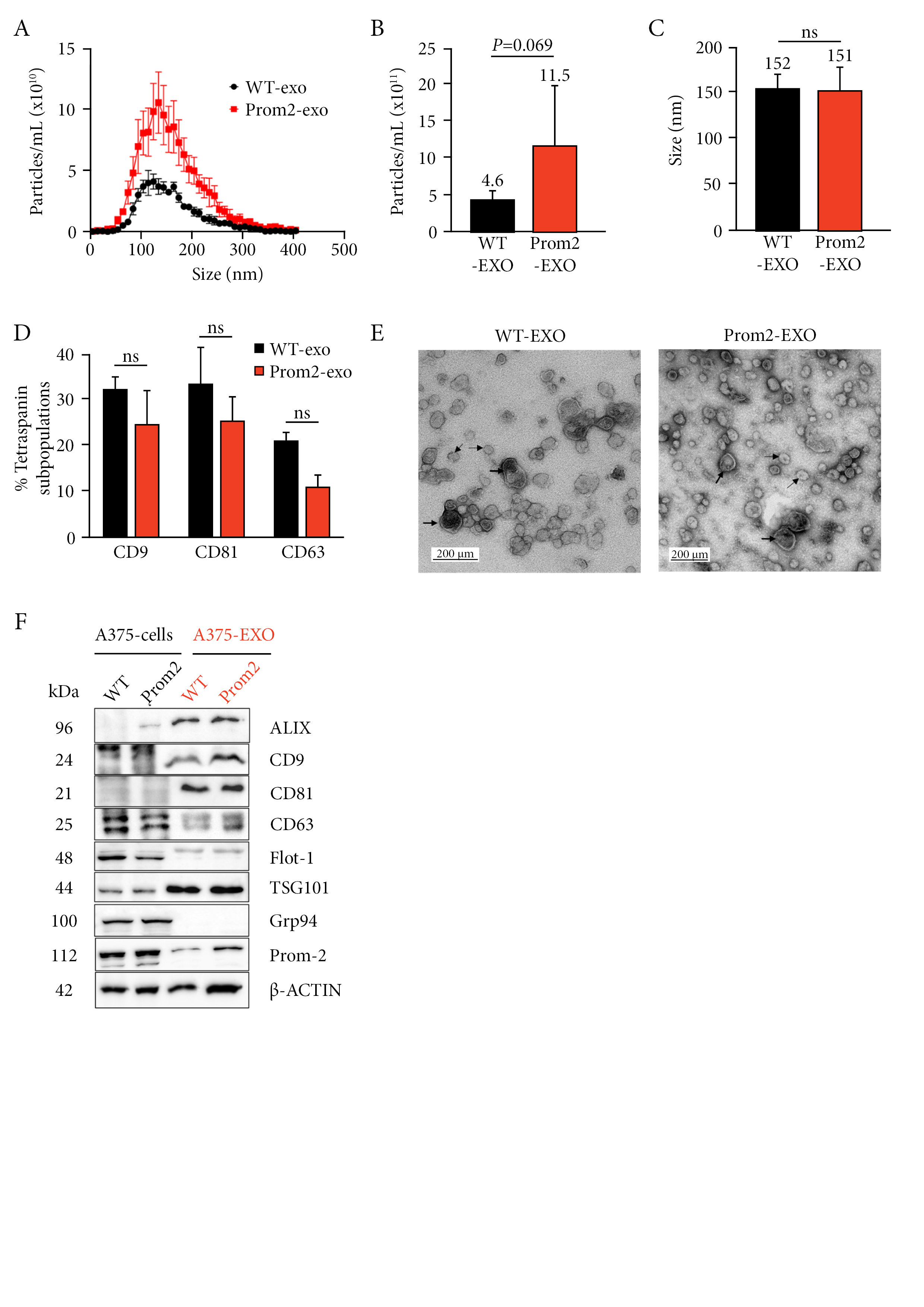

Supplement: Supplementary file 9 — Supporting information [file CTM2-14-e1632-s013.jpg]

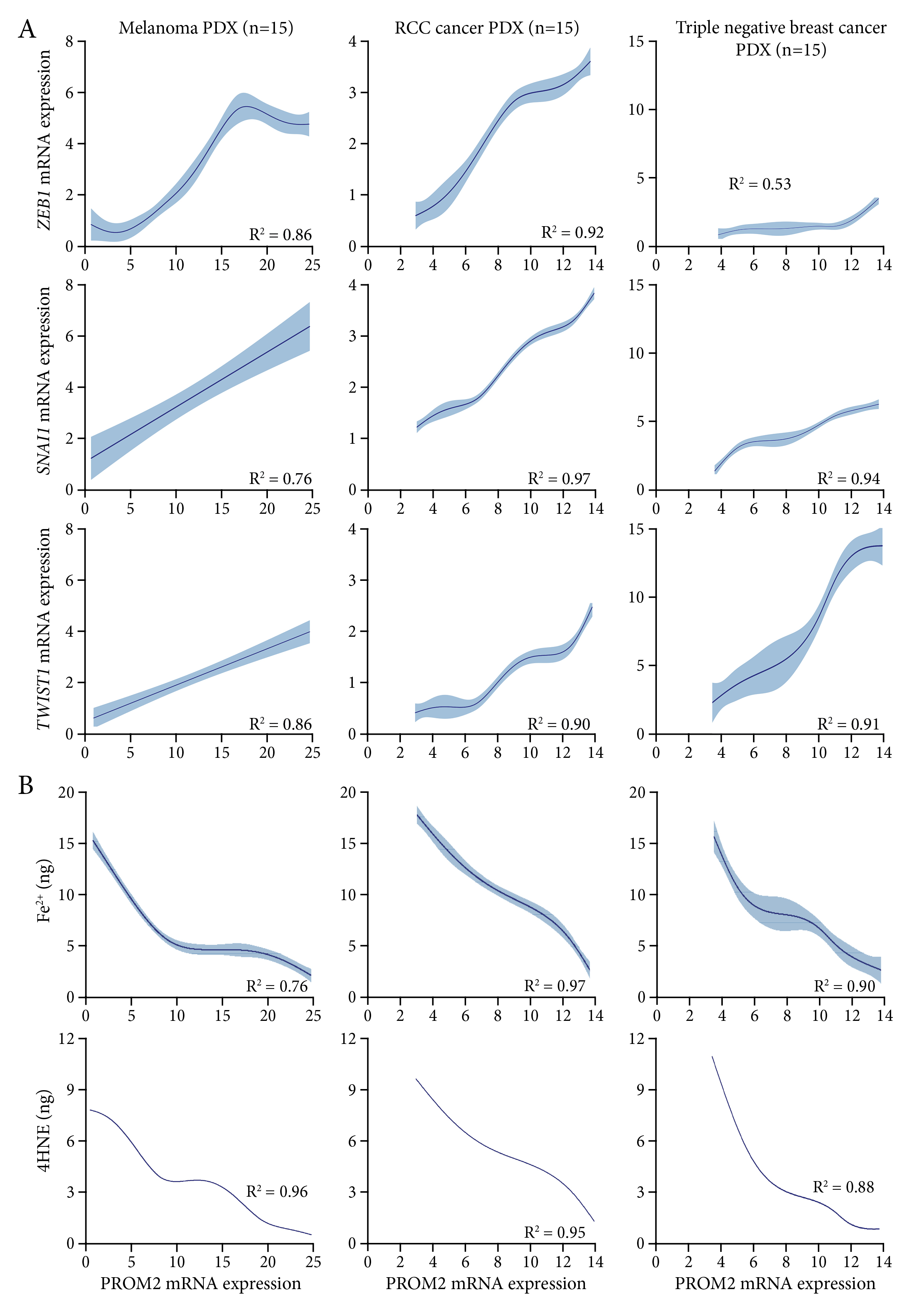

Supplement: Supplementary file 10 — Supporting information [file CTM2-14-e1632-s009.jpg]

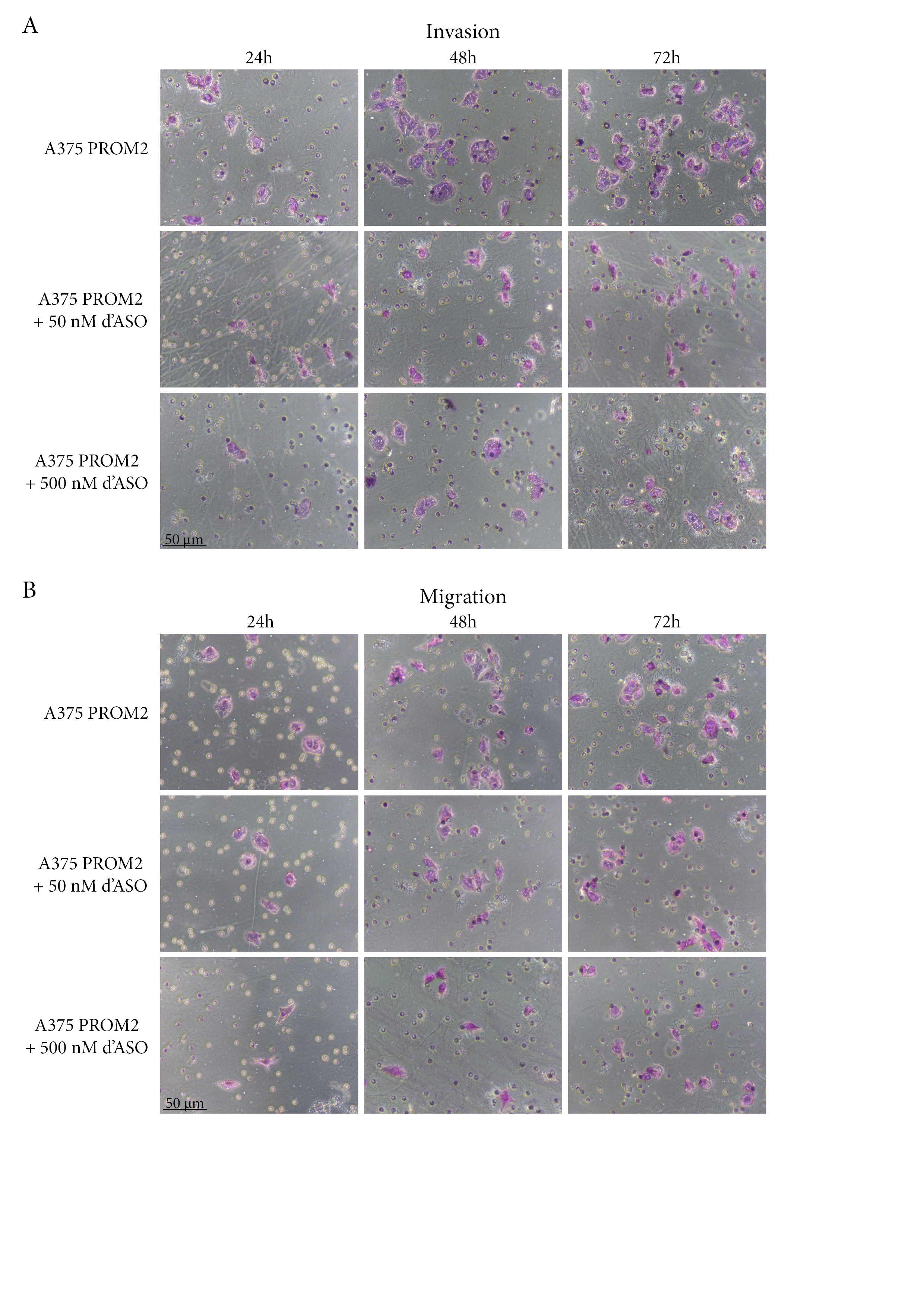

Supplement: Supplementary file 11 — Supporting information [file CTM2-14-e1632-s012.jpg]
