# Supplementary material for: PROM2 overexpression induces metastatic potential through epithelial‐to‐mesenchymal transition and ferroptosis resistance in human cancers
Source: Clin Transl Med. 2024 Mar 21;14(3):e1632. doi: 10.1002/ctm2.1632 (PMC10958126; doi:10.1002/ctm2.1632)
Supplement: Supplementary file 12 — Supporting information [file CTM2-14-e1632-s008.docx]

**Supplementary Figure 1.**

Diagram of the different methods used in this study.

**Supplementary Figure 2.**

Cell viability of A375 KO, A375 and A375 PROM2 cell lines treated for 24h with increasing concentrations of RSL3, Erastin or DFO.

**Supplementary Figure 3.**

**(A)** mRNA expression level of *PROM2* in Sk-Mel-28 KO (Cq > 40), Sk-Mel-28 and Sk-Mel-28 PROM2 cell lines (****P* < 0.001). **(B)** PROM2 immunofluorescence staining (in green, magnification X400) and protein normalized expression using western blot (***P* < 0.01). **(C,D)** Count of invading (C) or migrating (D) cells at 24, 48 and 72 hours in Boyden chamber (****P* < 0.001). **(E)** Histological sections of heart metastases and myocardial invasion in two different mice injected with Sk-Mel-28 PROM2 cell line. **(F)** Left panel shows histological sections of lungs from mice injected with 5.10^6^ Sk-Mel-28 or Sk-Mel-28 PROM2 cell lines. Right panel shows the mean surface area in percentage of lung metastases/total lung surface area quantified 8 weeks after injection (****P* < 0.001).

**Supplementary Figure 4.**

Images of the expansion of spheroids obtained from A375 KO, A375, and A375 PROM2 cell lines.

**Supplementary Figure 5.**

**(A)** Proliferation rate of A375 KO (white), A375 (grey) and A375 PROM2 (black) cell lines in vitro (percentage of viable cells) and after sub-cutaneous xenograft in mice (percentage of tumor volume). **(B)** Proliferation rate of Sk-Mel-28 KO, Sk-Mel-28 and Sk-Mel-28 PROM2 cell lines in vitro (percentage of viable cells) and after sub-cutaneous xenograft in mice (percentage of tumor volume). **(C)** Left panel shows *PROM2* mRNA expression in five patient-derived melanoma xenograft models (****P* < 0.001). The median (-ΔCq) for *PROM2* mRNA expression at 12 (dash-dotted line) distinguishes two groups of PDXs (< 12 = Low expression, PROM2-; ≥ 12 = High expression, PROM2+). Right panel shows PROM2 protein expression using immunohistochemistry (IHC) score in five patient-derived melanoma xenograft models (**P* < 0.05). (**D**) Left panel shows the relationship between tumor volume and day of euthanasia; right panel shows the absence of correlation between tumor volume doubling time and *PROM2* mRNA expression (*P* = 0.72).

**Supplementary Figure 6.**

**(A)** mRNA expression level of epithelial marker (*CDH1*), EMT markers (*ZEB1, ZEB2, SNAI1, SNAI2, TWIST1, TWIST2*) and mesenchymal marker (*VIM*) in Sk-Mel-28 KO, Sk-Mel-28 and Sk-Mel-28 PROM2 cell lines (****P* < 0.001). **(B)** Immunoblotting and quantification of protein level of ZEB1 and SNAIL1 in Sk-Mel-28 KO, Sk-Mel-28 and Sk-Mel-28 PROM2 cell lines (***P* < 0.01).

**Supplementary Figure 7.**

**(A)** Left panel shows mRNA expression of epithelial marker (*CDH1*, ****P* < 0.001), EMT markers (*ZEB1*, ****P* < 0.001; *ZEB2*, ****P* < 0.001; *SNAI1*, ***P* < 0.01; *SNAI2*, ****P* < 0.001; *TWIST1*, ****P* < 0.001; *TWIST2*, ****P* < 0.001) and mesenchymal marker (*VIM*, ***P* < 0.01) in subcutaneous tumor xenografts derived from A375 and A375 PROM2 cell lines. Right panel shows ZEB1 and SNAIL immunostainings in subcutaneous tumor xenografts with corresponding quantification of positive cells (****P* < 0.001). **(B)** mRNA expression of epithelial marker (*CDH1*, ****P* < 0.001), EMT markers (*ZEB1*, ****P* < 0.001; *ZEB2*, ****P* < 0.001; *SNAI1*, ****P* < 0.001; *SNAI2*, ****P* < 0.001; *TWIST1*, ****P* < 0.001; *TWIST2*, ****P* < 0.001) and mesenchymal marker (*VIM*, ****P* < 0.001) in subcutaneous tumor xenografts derived from Sk-Mel-28 and Sk-Mel-28 PROM2 cell lines. **(C)** mRNA expression level of epithelial marker (*CDH1*, ***P* < 0.01), EMT markers (*ZEB1,* ****P* < 0.001; *ZEB2*, ****P* < 0.001; *SNAI1*, ****P* < 0.001; *SNAI2*, ***P* < 0.01; *TWIST1*, ****P* < 0.001; *TWIST2*, ****P* < 0.001) and mesenchymal marker (*VIM*, ****P* < 0.001) in patient-derived melanoma xenografts expressing PROM2: low (PROM2-) or high (PROM2+).

**Supplementary Figure 8.**

**(A)** Quantification of Fe^2+^ (ng) in Sk-Mel-28 KO, Sk-Mel-28 and Sk-Mel-28 PROM2 cell lines without treatment, or after 24h of treatment with deferoxamine (DFO, ferroptosis inhibitor), or with RSL3 or Erastin (ferroptosis activators) (****P* < 0.001). **(B)** Cell viability of Sk-Mel-28 KO, Sk-Mel-28 and Sk-Mel-28 PROM2 cell lines after the addition of DFO, RSL3 or Erastin during 24 hours (****P* < 0.001). **(C)** Quantification of Fe^2+^ (box plot) and cell viability (dotted-dashed line) of Sk-Mel-28 PROM2 cell line treated for 24h with increasing concentrations of RSL3 or Erastin.

**Supplementary Figure 9.**

**(A)** Nanosight Tracking Analysis (NTA) of WT-EXO and Prom2-EXO representing the particle size distribution of 50-200 nm, which define the range size of exosomes. **(B)** Total concentration of WT-EXO and Prom2-EXO determined by NTA (n=4). **(C)** Mean size of exosomes derived from both cell lines. **(D)** Relative percentage of tetraspanin (CD9, CD81, and CD63) subpopulations in the exosomal fraction of both cell lines. **(E)** Transmission electron micrograph of WT-EXO and Prom2-EXO. **(F)** Western blot analysis indicating the presence of positive exosome markers ALIX, CD9, CD81, CD63, Flot-1, and TSG101.

**Supplementary Figure 10.**

**(A)** Correlation between *PROM2* and EMT marker mRNA expression in xenografts derived from human melanoma (left column), human renal cell carcinoma (RCC, middle column) and human triple negative breast cancer (right column) (****P* < 0.001). **(B)** Correlation between *PROM2* mRNA expression and Fe^2+^ quantification (top panel) or 4HNE quantification (lipid peroxidation, bottom panel) in xenografts derived from human melanoma (left column), human renal cell carcinoma (RCC, middle column) and human triple negative breast cancer (right column) (****P* < 0.001).

**Supplementary Figure 11.**

**(A)** Images of invading A375 KO, A375 and A375 PROM2 cell lines at 24, 48 and 72 hours in Boyden chamber. **(B)** Images of migrating A375 KO, A375 and A375 PROM2 cell lines at 24, 48 and 72 hours in Boyden chamber.
