# Supplementary material for: PROM2 overexpression induces metastatic potential through epithelial‐to‐mesenchymal transition and ferroptosis resistance in human cancers
Source: Clin Transl Med. 2024 Mar 21;14(3):e1632. doi: 10.1002/ctm2.1632 (PMC10958126; doi:10.1002/ctm2.1632)
Supplement: Supplementary file 13 — Supporting information [file CTM2-14-e1632-s004.docx]

| Type of cancers | Samples | Expression in cancer | Patients survival | References |
| --- | --- | --- | --- | --- |
| Bone marrow | Tumor mRNA | Up | - | [1] |
| Kidney | Tumor mRNA | Up | Poor | [1, 2] |
| Lung | Tumor mRNA | Up | Poor | [1] |
| Pancreas | Tumor mRNA and proteins | Up | Poor | [3-5] |
| Melanoma | Metastasic lymph nodes mRNA and proteins | Up | Poor | [6] |
| Neuroblastoma | Tumor mRNA | Up | Poor | [7] |
| Lung | Tumor mRNA | Up | Poor | [8] |
| Endometrial | Tumor mRNA | Up | Poor | [9] |

1. Saha, S.K., S.M.R. Islam, K.S. Kwak, M.S. Rahman, and S.G. Cho, *PROM1 and PROM2 expression differentially modulates clinical prognosis of cancer: a multiomics analysis.* Cancer Gene Ther, 2020. **27**(3-4): p. 147-167.

2. Zhang, B., W. Chu, F. Wen, et al., *Dysregulation of Long Non-coding RNAs and mRNAs in Plasma of Clear Cell Renal Cell Carcinoma Patients Using Microarray and Bioinformatic Analysis.* Front Oncol, 2020. **10**: p. 559730.

3. Li, W., Y. Zhu, K. Zhang, et al., *PROM2 promotes gemcitabine chemoresistance via activating the Akt signaling pathway in pancreatic cancer.* Exp Mol Med, 2020. **52**(3): p. 409-422.

4. Du, Y., W. Jiang, S. Hou, Z. Chen, and W. Zhou, *A Novel Cuproptosis-Associated Gene Signature to Predict Prognosis in Patients with Pancreatic Cancer.* Biomed Res Int, 2023. **2023**: p. 3419401.

5. Wu, T., T.Y. Qian, R.J. Lin, et al., *Construction and validation of a m6A RNA methylation and ferroptosis-related prognostic model for pancreatic cancer by integrated bioinformatics analysis.* J Gastrointest Oncol, 2022. **13**(5): p. 2553-2564.

6. Nguyen, T.T., G. Gapihan, P. Tetu, et al., *Increased risk of brain metastases among patients with melanoma and PROM2 expression in metastatic lymph nodes.* Clin Transl Med, 2020. **10**(8): p. e198.

7. Chen, Y., Z. Li, Q. Cao, et al., *Ferroptosis-related gene signatures in neuroblastoma associated with prognosis.* Front Cell Dev Biol, 2022. **10**: p. 871512.

8. Tang, J., D. Shu, Z. Fang, and G. Yang, *Prominin 2 decreases cisplatin sensitivity in non-small cell lung cancer and is modulated by CTCC binding factor.* Radiol Oncol, 2023. **57**(3): p. 325-336.

9. Jiang, J., C. Zhang, J. Wang, et al., *Knockdown of PROM2 Enhances Paclitaxel Sensitivity in Endometrial Cancer Cells by Regulating the AKT/FOXO1 Pathway.* Anticancer Agents Med Chem, 2023. **23**(19): p. 2127-2134.
