# Supplementary material for: PROM2 overexpression induces metastatic potential through epithelial‐to‐mesenchymal transition and ferroptosis resistance in human cancers
Source: Clin Transl Med. 2024 Mar 21;14(3):e1632. doi: 10.1002/ctm2.1632 (PMC10958126; doi:10.1002/ctm2.1632)
Supplement: Supplementary file 14 — Supporting information [file CTM2-14-e1632-s005.docx]

**Statistical analysis**

**Supplementary data:**

*PROM2 in Sk-mel28 lines:* The *PROM2* mRNA expression was compared according to the cell lines (Sk-Mel-28 KO, Sk-Mel-28, and Sk-Mel-28 PROM2) using ANOVA test with multiple comparison of means, and was graphically presented as bar graph.

*In vitro invasion and migration:* The number of invasive cells and the number of migrating cells were compared as function of cell lines (Sk-Mel-28 KO, Sk-Mel-28, and Sk-Mel-28 PROM2), using ANOVA test with multiple comparison of means at 24, 48 and 72h, and was graphically presented as bar graph.

*Epithelial to mesenchymal transition:* the median gene expression (-dCq) of *CDH1*, *ZEB1-2*, *SNAI1-2*, *TWIST1-2*, and *VIM* was compared as function of cell lines (Sk-Mel-28 KO, Sk-Mel-28, and Sk-Mel-28 PROM2) using ANOVA test of comparison of means. Graphically, results were presented as boxplot.

*Metastatic potential:* lung metastatic area was compared as function of cell lines (Sk-Mel-28, and Sk-Mel-28 PROM2) using Student t test and was graphically presented as bar graph. Then, the mean values of gene expression (-dCq) *CDH1*, *ZEB1-2*, *SNAI1-2*, *TWIST1-2*, and *VIM* were compared according to the cell lines xenograft (A375, and A375 PROM2) using a Student t test. In addition, the % of positive cells for ZEB1 and SNAIL/SLUG was graphically presented as bar graph and was compared according to the *PROM2* mRNA overexpression status (PROM2 +/-) according to the cell lines xenograft (A375, and A375 PROM2) using a Student t test. Finally, the mean values of gene expression (-dCq) *CDH1*, *ZEB1-2*, *SNAI1-2*, *TWIST1-2*, and *VIM* were compared according to the cell lines xenograft (Sk-Mel-28, and Sk-Mel-28 PROM2) using a Student t test.

*Ferroptosis:* the mean values of intracellular ferrous iron were compared as function of cell lines (Sk-Mel-28 KO, Sk-Mel-28, and Sk-Mel-28 PROM2) and as function of activation of ferroptosis status (none, DFO, RSL3 and Erastin) using ANOVA test with multiple comparison of means, and was graphically presented as bar graph. The mean values of cell viability (%) were then compared as function of cell lines and as function of ferroptosis activating agents (none, DFO, RSL3 and Erastin) using ANOVA test with multiple comparison of means, and was graphically presented as bar graph. We then assessed the nature of the relationship between cell viability (%) and ferrous iron quantity (ng), and the doses of ferroptosis activating agents (RSL3 and Erastin) using a non-parametric regression via smoothing splines in Sk-Mel-28 PROM2 line.

**Quantitative Data**

**Fig 1A**

The mean number of invading cells was 5.8 ± 0.8, 10.0 ± 1.3, and 17.0 ± 1.2 at 24h for A375 KO, A375 and A375 PROM2 respectively (*P* < 0.0001 for A375 KO/A375, and for A375/A375 PROM2); 9.0 ± 1.1, 15.0 ± 1.3, and 23.0 ± 1.7 at 48h (*P* < 0.0001 for A375 KO/A375, and for A375/A375 PROM2); and 13.0 ± 1.3, 24.0 ± 1.5, and 31.0 ± 2.0 at 72h (*P* < 0.0001 for A375 KO/A375, and for A375/A375 PROM2).

**Fig 2B**

The mean number of migrating cells was 6.0 ± 1.5, 10.0 ± 1.2, and 16.0 ± 1.2 at 24h for A375 KO, A375 and A375 PROM2 respectively (*P* < 0.0001 for A375 KO/A375, and for A375/A375 PROM2); 13.0 ± 1.8, 19.0 ± 1.6, and 25.0 ± 1.5 at 48h (*P* < 0.0001 for A375 KO/A375, and for A375/A375 PROM2); and 20.0 ± 1.8, 28.0 ± 1.5, and 37.0 ± 1.6 at 72h (*P* < 0.0001 for A375 KO/A375, and for A375/A375 PROM2).

**Fig 2C**

The median gene expression (-dCq) of *CDH1* was 5.9 [5.6-6.1], 3.9 [3.5-4.2], and 1.0 [0.9-1.0] for A375 KO, A375, and A375 PROM2 respectively (*P* < 0.01); for *ZEB1* it was 1.0 [0.9-1.0], 3.0 [2.9-3.1], and 5.4 [5.0-5.7] respectively (*P* < 0.01); for *ZEB2* it was 0.4 [0.2-0.5], 1.2 [1.1-1.3], and 2.0 [1.9-2.1] respectively (*P* < 0.01); for *SNAI1* it was 0.6 [0.5-0.7], 2.3 [2.2-2.4], and 5.0 [4.9-5.1] respectively (*P* < 0.01); for *SNAI2* it was 1.0 [0.9-1.0], 2.0 [1.9-2.0], and 2.6 [2.5-2.6] respectively (*P* < 0.01); for *TWIST1* it was 1.6 [1.5-1.6], 3.1 [2.9-3.2], and 5.8 [5.7-5.9] respectively (*P* < 0.01); for *TWIST2* it was 0.5 [0.4-0.5], 2.7 [2.5-2.8], and 8.7 [8.5-9.0] respectively (*P* < 0.01); and for *VIM* it was 0.9 [0.8-0.9], 2.7 [2.6-2.7], and 5.0 [4.9-5.1] respectively (*P* < 0.01).

**Fig 2A**

The mean value of lung metastatic area was 4.4% ± 1.5 and 61.2% ± 6.5 for A375 and A375 PROM2 respectively (*P* < 0.0001).

**Fig 2B**

The mean value of *ZEB1* expression was 1.0 ± 0.03 and 9.1 ± 0.8 for A375 and A375 PROM2 respectively in the xenograft group (*P* < 0.0001); 0.9 ± 0.1 and 44.5 ± 2.1 respectively in the lung metastases group (*P* < 0.0001).

The mean value of *SNAI1* expression was 1.1 ± 0.09 and 6.7 ± 0.0 for A375 and A375 PROM2 respectively in the xenograft group (*P* = 0.01); 1.3 ± 0.2 and 52.0 ± 7.0 respectively in the lung metastases group (*P* < 0.001).

The mean value of *TWIST1* expression was 1.1 ± 0.2 and 8.1 ± 0.5 for A375 and A375 PROM2 respectively in the xenograft group (*P* < 0.0001); 1.0 ± 0.1 and 21.5 ± 2.1 respectively in the lung metastases group (*P* < 0.001).

**Fig 2D**

The mean values of gene expression (-dCq) of *CDH1* were 4.8 ± 3.8 and 14.3 ± 1.5 for PROM2+ and PROM2- status respectively (*P* < 0.01).

The mean values of gene expression (-dCq) of *ZEB1* were 4.7 ± 0.7 and 0.2 ± 0.1 for PROM2+ and PROM2- status respectively (*P* < 0.0001).

The mean values of gene expression (-dCq) of *ZEB2* were 1.3 ± 0.2 and 0.2 ± 0.1 for PROM2+ and PROM2- status respectively (*P* < 0.0001).

The mean values of gene expression (-dCq) of *SNAI1* were 5.6 ± 0.9 and 2.3 ± 1.2 for PROM2+ and PROM2- status respectively (*P* < 0.001).

The mean values of gene expression (-dCq) of *SNAI2* were 2.7 ± 1.1 and 0.6 ± 0.3 for PROM2+ and PROM2- status respectively (*P* < 0.01).

The mean values of gene expression (-dCq) of *TWIST1* were 2.7 ± 0.6 and 0.8 ± 0.4 for PROM2+ and PROM2- status respectively (*P* < 0.001).

The mean values of gene expression (-dCq) of *TWIST2* were 7.7 ± 0.8 and 1.5 ± 0.8 for PROM2+ and PROM2- status respectively (*P* < 0.0001).

The mean values of gene expression (-dCq) of *VIM* were 2.8 ± 0.4 and 1.1 ± 0.6 for PROM2+ and PROM2- status respectively (*P* < 0.001).

**Fig 2E**

The mean values of % of ZEB1 positive cells were 11.0% ± 1.1 and 62.0% ± 3.0 for PROM2-/PROM2 + respectively (*P* < 0.0001).

The mean values of % of SNAIL/SLUG positive cells were 28.0% ± 2.1 and 63.0% ± 3.4 for PROM2-/PROM2 + respectively (*P* < 0.0001).

**Fig 3A**

The mean values of intracellular ferrous iron (ng) were 0.70 ± 0.04, 0.66 ± 0.01, 0.82 ± 0.005, and 0.85 ± 0.005 for none treatment, DFO, RSL3, and Erastin respectively in A375 KO cell line (*P* < 0.0001).

The mean values of intracellular ferrous iron (ng) were 0.64 ± 0.02, 0.59 ± 0.005, 0.76 ± 0.03, and 0.76 ± 0.005 for none treatment, DFO, RSL3, and Erastin respectively in A375 cell line (*P* < 0.0001).

The mean values of intracellular ferrous iron (ng) were 0.52 ± 0.03, 0.45 ± 0.01, 0.49 ± 0.01, and 0.47 ± 0.01 for none treatment, DFO, RSL3, and Erastin respectively in A375 PROM2 cell line (*P* < 0.0001).

**Fig 3B**

The mean values of cell viability (%) were 52.0% ± 2.0, 70.0% ± 1.0, and 99.0% ± 1.1 for A375 KO, A375, and A375 PROM2 respectively in RSL3 group (*P* < 0.0001).

The mean values of cell viability (%) were 50.0% ± 2.0, 70.0% ± 3.0, and 99.0% ± 0.6 for A375 KO, A375, and A375 PROM2 respectively in Erastin group (*P* < 0.0001).

**Fig 3C**

The mean values of intracellular ferrous iron (ng) were 2.0 ± 0.2, 1.9 ± 0.2, 1.8 ± 0.1, 2.0 ± 0.2, 2.2 ± 0.2, and 2.4 ± 0.1 for 0.0, 0.5, 1.0, 1.5, 2.0, and 2.5 μg of RSL3 respectively in A375PROM2 cell line (P < 0.01).

The mean values of cell viability (%) were 99.6 ± 0.5, 99.3 ± 1.1, 99.6 ± 0.6, 95.0 ± 1.0, 90.0 ± 2.0, and 80.0 ± 1.0 for 0.0, 0.5, 1.0, 1.5, 2.0, and 2.5 μg of RSL3 respectively in A375PROM2 cell line (P < 0.0001).

The mean values of intracellular ferrous iron (ng) were 2.0 ± 0.2, 1.8 ± 0.2, 1.6 ± 0.1, 1.5 ± 0.2, 2.0 ± 0.2, and 2.4 ± 0.1, for 0.0, 5.0, 10.0, 15.0, 20.0, and 25.0 μg of ERASTIN respectively in A375PROM2 cell line (P < 0.001).

The mean values of cell viability (%) were 100.0 ± 0.0, 100.0 ± 0.0, 99.6 ± 0.6, 99.6 ± 0.6, 85.0 ± 2.5, and 60.0 ± 1.0 for 0.0, 5.0, 10.0, 15.0, 20.0, and 25.0 μg of ERASTIN respectively in A375PROM2 cell line (P < 0.0001).

**Fig 3F**

The mean values of cell ferrous iron quantities (ng) were for 0.53 ± 0.01, 0.5 ± 0.01, 0.66 ± 0.01, 0.65 ± 0.01, and 0.67 ± 0.01for XM1 to XM5 models respectively. Except for XM3 and XM4 (*P* = 0.5)), and XM3 and XM5 (*P* = 0.7), there was a significant difference between models (*P* < 0.0001).

**Fig 3G**

The mean values of 4HNE were 31.0 ± 0.2, 19.6 ± 0.2, and 13.2 ± 0.2 for A375 KO, A375, and A375 PROM2 cell lines respectively (*P* < 0.0001 for A375 KO/A375, and for A375/A375 PROM2).

R2 = 0.95, P < 0.0001.

**Fig 4B-C**

The mean number of invading cells was 8.0 ± 1.0, 5.3 ± 0.6, and 2.0 ± 1.0 at 24h for A375 PROM2, A375 PROM2 treated with 50µM of ASO, and A375 PROM2 treated with 500µM of ASO respectively (*P* < 0.0001); 16.0 ± 0.6, 12.0 ± 1.0, and 9.0 ± 0.6 at 48h (P < 0.0001); and 26.0 ± 1.5, 17.0 ± 1.0, and 12.0 ± 1.1 at 72h (*P* < 0.0001).

The mean number of migrating cells was 11.0 ± 1.0, 6.0 ± 0.6, and 3.0 ± 1.0 at 24h for A375 PROM2, A375 PROM2 treated with 50µM of ASO, and A375PROM2 treated with 500µM of ASO respectively (*P* < 0.0001); 25.0 ± 2.0, 15.0 ± 1.1 and 11.0 ± 1.0 at 48h (*P* < 0.0001); and 40.0 ± 3.0, 22.0 ± 3.0, and 15.0 ± 1.5 at 72h (*P* < 0.0001).

**Fig 4D**

The mean values of intracellular ferrous iron (ng) were 3.9 ± 0.0, 1.71 ± 0.0, 5.2 ± 0.07, 7.0 ± 0.07, and 7.5 ± 0.07 for A375, A375 PROM2, A375 PROM2 treated with 50µM of ASO, A375 PROM2 treated with 500µM of ASO and A375 KO (*P* < 0.0001).

**Fig 4E**

The median gene expression (-dCq) of *CDH1* was 0.5 [0.4-0.6], and 2.7 [2.7-2.8] for A375 PROM2, and A375 PROM2 treated with 50µM of ASO respectively (*P* =0.04); for *ZEB1* it was 3.0 [2.9-3.0], and 0.3 [0.2-0.3] respectively (*P* = 0.04); for *ZEB2* it was 0.3 [0.2-0.3], and 0.1 [0.0-0.1] respectively (*P* = 0.07); for *SNAI1* it was 2.8 [2.7-2.8], and 0.4 [0.3-0.4] respectively (*P* = 0.04); for *SNAI2* it was 0.6 [0.5-0.6], and 0.2 [0.1-0.2] respectively (*P* = 0.04); for *TWIST1* it was 3.0 [2.9-3.1], and 0.8 [0.7-0.8] respectively (*P* = 0.04); for *TWIST2* it was 8.6 [8.5-8.7], and 2.8 [2.7-2.8] respectively (*P* = 0.04); and for *VIM* it was 2.8 [2.8-2.9], and 0.4 [0.3-0.4] respectively (*P* = 0.04).

**Fig 4F**

The mean value of lung metastatic area was 4.4% ± 1.5, 61.0% ± 6.5, 9.0% ± 1.3, and 7.0% ± 0.8 for A375, A375 PROM2, A375 PROM2 treated with 50µM of ASO, and A375 PROM2 treated with 500µM of ASO respectively. Significant differences were found between A375 and A375 PROM2 (*P* <0.001), between A375 PROM2 and A375 PROM2 treated with 50µM of ASO (*P* < 0.001), and between A375 PROM2 and A375 PROM2 treated with 500µM of ASO (*P* < 0.001). No significant differences were found between A375 and A375 PROM2 treated with 50µM (*P* = 0.15) or 500µM of ASO (*P* = 0.7), and between A375 PROM2 treated with 50µM or 500µM of ASO (*P* = 0.7).

**Fig 5A**

The mean value of *PROM2* mRNA expression was 1.0 ± 0.1, 3.2 ± 0.0, 4.0 ± 0.0, and 4.6 ± 0.0 for T0, T30, T60, and T120 respectively (*P* < 0.0001).

**Fig 5B**

The mean value of *PROM2* gene expression/*GAPDH* ratio was 5.3 ± 0.3, 10.0 ± 1.3, 15.0 ± 0.5, and 45.0 ± 3.0 for A375, A375 PROM2, A375 PROM2 xenograft, and A375 PROM2 lung metastasis respectively (*P* < 0.0001).

**Fig 5D**

The mean number of metastases was 3.8 ± 0.8, and 7.4 ± 1.1 for passage 1 and 2 respectively (P < 0.0001).

**Fig 5E**

The mean value of *PROM2* mRNA expression (-dCq) was 3.5 ± 0.1, 4.5 ± 0.1, and 5.8 ± 0.1 for passage 1, 2, and 3 respectively (*P* < 0.001)

The mean value of *CAV1* mRNA expression (-dCq) was 1.8 ± 0.1, 2.8 ± 0.0, and 3.9 ± 0.1 for passage 1, 2, and 3 respectively (*P* < 0.001)

The mean value of *CDH1* mRNA expression (-dCq) was 0.7 ± 0.1, 0.4 ± 0.1, and 0.2 ± 0.0 for passage 1, 2, and 3 respectively (*P* = 0.02)

The mean value of *ZEB1* mRNA expression (-dCq) was 1.7 ± 0.0, 2.3 ± 0.1, and 3.2 ± 0.1 for passage 1, 2, and 3 respectively (*P* = 0.001)

The mean value of *ZEB2* mRNA expression (-dCq) was 1.4 ± 0.0, 1.6 ± 0.1, and 2.7 ± 0.1 for passage 1, 2, and 3 respectively (*P* < 0.01)

The mean value of *SNAI1* mRNA expression (-dCq) was 1.3 ± 0.1, 1.8 ± 0.1, and 2.4 ± 0.0 for passage 1, 2, and 3 respectively (*P* < 0.01)

The mean value of *SNAI2* mRNA expression (-dCq) was 2.2 ± 0.1, 3.1 ± 0.1, and 4.3 ± 0.1 for passage 1, 2, and 3 respectively (*P* < 0.01)

The mean value of *TWIST1* mRNA expression (-dCq) was 1.5 ± 0.1, 2.5 ± 0.1, and 4.1 ± 0.1 for passage 1, 2, and 3 respectively (*P* < 0.001)

The mean value of *TWIST2* mRNA expression (-dCq) was 1.6 ± 0.1, 2.4 ± 0.1, and 3.8 ± 0.1 for passage 1, 2, and 3 respectively (*P* < 0.001)

The mean value of *VIM* mRNA expression (-dCq) was 1.1 ± 0.0, 1.5 ± 0.1, and 2.1 ± 0.1 for passage 1, 2, and 3 respectively (*P* < 0.01)

**Suppl 1A**

The mean *PROM2* mRNA expression was 0.28 ± 0.19, 1.71 ± 0.07 and 7.11 ± 0.67 for A375 KO, A375 and A375 PROM2 respectively (*P* = 0.001 for A375 KO/A375, and *P* < 0.001 for A375/A375 PROM2)

**Suppl 1D**

The median *PROM2* mRNA expression was 20.0 [12.0-24.0], 19.0 [14.0-22.0], 7.9 [5.9-8.1], 7.9 [6.6-8.2], and 5.7 [4.6-6.4], for XM1, XM2, XM3, XM4, and XM5 respectively (*P* < 0.0001).

The median PROM2 immunochemistry score was 5.8 [5.8-6.6], 7.4 [6.8-7.6], 2.4 [1.8-2.6], 1.2 [0.8-1.8], and 0.8 [0.6-0.8], for XM1, XM2, XM3, XM4, and XM5 respectively (*P* = 0.01).

Table of medians under the graph with medians.

R2 = 0.007 (*P* = 0.72)

**Suppl 2A:**

The mean values of *PROM2* mRNA expression were 0.2 ± 0.2, 1.9 ± 0.0, and 5.9 ± 0.6 for Sk-Mel-28 KO, Sk-Mel-28, and Sk-Mel-28 PROM2 respectively (*P* < 0.0001)

**Suppl 3A**

The mean number of invading cells was 4.4 ± 0.9, 9.1 ± 1.3, and 14.0 ± 1.2 at 24h for Sk-Mel-28 KO, Sk-Mel-28, and Sk-Mel-28 PROM2 respectively (*P* < 0.0001 for Sk-Mel-28 KO/Sk-Mel-28, and for Sk-Mel-28/Sk-Mel-28 PROM2); 8.2 ± 1.3, 13.1 ± 1.1, and 20.0 ± 1.6 at 48h (*P* < 0.0001 for Sk-Mel-28 KO/Sk-Mel-28, and for Sk-Mel-28/Sk-Mel-28 PROM2); and 12.3 ± 1.5, 22.0 ± 1.4, and 29.0 ± 2.0 at 72h (*P* < 0.0001 for Sk-Mel-28 KO/Sk-Mel-28, and for Sk-Mel-28/Sk-Mel-28 PROM2).

**Suppl 3B**

The mean number of migrating cells was 5.0 ± 1.3, 10.0 ± 1.4, and 14.0 ± 1.2 at 24h for Sk-Mel-28 KO, Sk-Mel-28, and Sk-Mel-28 PROM2 respectively (*P* < 0.0001 for Sk-Mel-28 KO/Sk-Mel-28, and for Sk-Mel-28/Sk-Mel-28 PROM2); 11.0 ± 1.1, 16.0 ± 1.0, and 24.0 ± 1.5 at 48h (*P* < 0.0001 for Sk-Mel-28 KO/Sk-Mel-28, and for Sk-Mel-28/Sk-Mel-28 PROM2); and 18.0 ± 1.4, 28.0 ± 1.4, and 34.0 ± 1.0 at 72h (*P* < 0.0001 for Sk-Mel-28 KO/Sk-Mel-28, and for Sk-Mel-28/Sk-Mel-28 PROM2).

**Suppl 3C**

The mean value of gene expression (-dCq) of *CDH1* was 5.8 ± 0.3, 3.3 ± 0.2, and 1.0 ± 0.1 for Sk-Mel-28 KO, Sk-Mel-28, and Sk-Mel-28 PROM2 respectively (*P* < 0.0001); for *ZEB1* it was 0.9 ± 0.1, 2.2 ± 0.1, and 6.0 ± 0.1 respectively (*P* < 0.0001); for *ZEB2* it was 0.3 ± 0.1, 1.2 ± 0.1, and 2.0 ± 0.1 respectively (*P* < 0.0001); for *SNAI1* it was 0.9 ± 0.1, 2.9 ± 0.2, and 5.4 ± 0.3 respectively (*P* < 0.0001); for *SNAI2* it 0.1 ± 0.0, 1.6 ± 0.1, and 2.1 ± 0.1 respectively (*P* < 0.0001); for *TWIST1* it was 1.7 ± 0.1, 2.3 ± 0.2, and 5.8 ± 0.1 respectively (*P* < 0.0001); for *TWIST2* it was 1.2 ± 0.5, 5.0 ± 0.2, and 8.3 ± 0.6 respectively (*P* < 0.0001); and for *VIM* it 0.7 ± 0.1, 3.0 ± 0.5, and 5.3 ± 0.1 respectively (*P* < 0.0001).

**Suppl 4B**

The mean value of lung metastatic area was 7.0% ± 1.0 and 52.0% ± 5.0 for Sk-Mel-28, and Sk-Mel-28 PROM2 respectively (*P* < 0.0001)

**Suppl 4C**

The mean values of gene expression (-dCq) of *CDH1* were 4.3 ± 0.3, and 0.9 ± 0.1 for A375, and A375 PROM2 xenograft respectively (*P* < 0.001).

The mean values of gene expression (-dCq) of *ZEB1* were 0.9 ± 0.1, and 4.4 ± 0.4 for A375, and A375 PROM2 xenograft respectively (*P* < 0.001).

The mean values of gene expression (-dCq) of *ZEB2* were 0.4 ± 0.1, and 1.9 ± 0.1 for A375, and A375 PROM2 xenograft respectively (*P* < 0.0001).

The mean values of gene expression (-dCq) of *SNAI1* were 1.9 ± 0.2, and 6.9 ± 0.9 for A375, and A375 PROM2 xenograft respectively (*P* < 0.01).

The mean values of gene expression (-dCq) of *SNAI2* were 1.2 ± 0.1, and 7.0 ± 0.4 for A375, and A375 PROM2 xenograft respectively (*P* < 0.0001).

The mean values of gene expression (-dCq) of *TWIST1* were 1.8 ± 0.2, and 5.9 ± 0.5for A375, and A375 PROM2 xenograft respectively (*P* < 0.001).

The mean values of gene expression (-dCq) of *TWIST2* were 1.8 ± 0.3 and 5.3 ± 0.3 for A375, and A375 PROM2 xenograft respectively (*P* < 0.0001).

The mean values of gene expression (-dCq) of *VIM* were 1.2 ± 0.1 and 4.7 ± 0.7 for A375, and A375 PROM2 xenograft respectively (*P* < 0.01).

**Suppl 4C**

The mean values of % of ZEB1 positive cells were 6.0% ± 0.8 and 77.0% ± 4.0 for A375 and A375 PROM2 respectively (*P* < 0.0001).

The mean values of % of SNAIL/SLUG positive cells were 2.0% ± 0.8 and 83.0% ± 3.0 for A375 and A375 PROM2 respectively (*P* < 0.0001).

**Suppl 4D**

The mean values of gene expression (-dCq) of *CDH1* were 4.9 ± 0.5, and 1.8 ± 0.3 for Sk-Mel-28, and Sk-Mel-28 PROM2 xenograft respectively (*P* < 0.001).

The mean values of gene expression (-dCq) of *ZEB1* were 1.2 ± 0.4, and 5.5 ± 0.2 for Sk-mel28, and Sk-Mel-28 PROM2 xenograft respectively (*P* < 0.0001).

The mean values of gene expression (-dCq) of *ZEB2* were 0.1 ± 0.1, and 1.6 ± 0.1 for Sk-mel28, and Sk-Mel-28 PROM2 xenograft respectively (*P* < 0.0001).

The mean values of gene expression (-dCq) of *SNAI1* were 1.2 ± 0.4, and 8.0 ± 1.3 for Sk-Mel-28, and Sk-Mel-28 PROM2 xenograft respectively (*P* < 0.001).

The mean values of gene expression (-dCq) of *SNAI2* were 1.0 ± 0.2, and 6.3 ± 0.4 for Sk-Mel-28, and Sk-Mel-28 PROM2 xenograft respectively (*P* < 0.0001).

The mean values of gene expression (-dCq) of *TWIST1* were 1.1 ± 0.3, and 6.6 ± 0.5 for Sk-Mel-28, and Sk-Mel-28 PROM2 xenograft respectively (*P* < 0.0001).

The mean values of gene expression (-dCq) of *TWIST2* were 1.6 ± 0.3 and 4.2 ± 0.3 for Sk-Mel-28, and Sk-Mel-28 PROM2 xenograft respectively (*P* < 0.0001).

The mean values of gene expression (-dCq) of *VIM* were 0.2 ± 0.1 and 5.4 ± 0.7 for Sk-Mel-28, and Sk-Mel-28 PROM2 xenograft respectively (*P* < 0.001).

**Suppl 5A**

The mean values of intracellular ferrous iron (ng) for none treatment were 6.0 ± 0.2, 4.6 ± 0.02, and 2.0 ± 0.01, for Sk-Mel-28 KO, Sk-Mel-28, and Sk-Mel-28 PROM2 respectively (*P* < 0.001 for Sk-Mel-28 KO/Sk-Mel-28, and for Sk-Mel-28/Sk-Mel-28 PROM2).

The mean values of intracellular ferrous iron (ng) for treatment by DFO were 5.3 ± 0.1, 3.6 ± 0.0, and 1.3 ± 0.1, for Sk-Mel-28 KO, Sk-Mel-28, and Sk-Mel-28 PROM2 respectively (*P* < 0.0001 for Sk-Mel-28 KO/Sk-Mel-28, and for Sk-Mel-28/Sk-Mel-28 PROM2).

The mean values of intracellular ferrous iron (ng) for treatment by RSL3 were 7.5 ± 0.1, 6.0 ± 0.1, and 2.1 ± 0.2, for Sk-Mel-28 KO, Sk-Mel-28, and Sk-Mel-28 PROM2 respectively (*P* < 0.0001 for Sk-Mel-28 KO/Sk-Mel-28, and for Sk-Mel-28/Sk-Mel-28 PROM2).

The mean values of intracellular ferrous iron (ng) for treatment by Erastin were 7.3 ± 0.1, 6.0 ± 0.1, and 1.7 ± 0.1, for Sk-Mel-28 KO, Sk-Mel-28, and Sk-Mel-28 PROM2 respectively (*P* < 0.0001 for Sk-Mel 28 KO/Sk-Mel-28, and for Sk-Mel-28/Sk-Mel 28 PROM2).

**Suppl 5B**

The mean values of cell viability (%) were 100% ± 0.0 for the three lines Sk-Mel-28 KO, Sk-Mel-28, and Sk-Mel-28 PROM2 in none treatment group with no significant differences (*P* ≥ 0.05).

The mean values of cell viability (%) for treatment by DFO were 100% ± 0.6; 99% ± 1.5, and 100% ± 0.6 for Sk-Mel-28 KO, Sk-Mel-28, and Sk-Mel-28 PROM2 respectively with no significant differences (*P* ≥ 0.05).

The mean values of cell viability (%) for treatment by RSL3 were 50% ± 2.1; 79% ± 5.0, and 100% ± 1.0 for Sk-Mel-28 KO, Sk-Mel-28, and Sk-Mel-28 PROM2 respectively (*P* < 0.001 for Sk-Mel-28 KO/Sk-Mel-28, and for Sk-Mel-28/Sk-Mel-28 PROM2).

The mean values of cell viability (%) for treatment by Erastin were 46% ± 2.0; 71% ± 1.0, and 99% ± 1.0 for Sk-Mel-28 KO, Sk-Mel-28, and Sk-Mel-28 PROM2 respectively (*P* < 0.0001 for Sk-Mel-28 KO/Sk-Mel-28, and for Sk-Mel-28/Sk-Mel-28 PROM2).

**Suppl 6A**

*Melanoma:*

ZEB1 R2 = 0.86, P<0.0001

SNAI1 R2 = 0.76, P<0.0001

TWIST R2 = 0.86, P<0.0001

*Renal cell carcinoma:*

ZEB1 R2 = 0.92, P<0.0001

SNAI1 R2 = 0.97, P<0.0001

TWIST R2 = 0.9, P<0.0001

*Breast cancer:*

ZEB1 R2 = 0.49, P<0.001

SNAI1 R2 = 0.94, P<0.0001

TWIST R2 = 0.9, P<0.0001

**Suppl 6B**

*Ferrous iron:*

Melanoma R2 = 0.73, P < 0.0001

Renal cell Carcinoma R2 = 0.97, P < 0.0001

Breast cancer R2 = 0.9, P < 0.0001

*4HNE:*

Melanoma R2 = 0.96, P < 0.0001

Renal cell carcinoma R2 = 0.95, P < 0.0001

Breast cancer R2 = 0.88, P< 0.0001
